# Patients’ Experiences of Digital Health Interventions for the Self-Management of Chronic Pain: Systematic Review and Thematic Synthesis

**DOI:** 10.2196/69100

**Published:** 2025-03-18

**Authors:** Ashleigh Main, Haruno McCartney, Maryam Ibrar, Fiona Muirhead, Alexandra Mavroeidi, Harleen Kaur Rai, Roma Maguire

**Affiliations:** 1 Department of Computer and Information Sciences University of Strathclyde Glasgow United Kingdom; 2 Physical Activity for Health School of Psychological Sciences and Health University of Strathclyde Glasgow United Kingdom; 3 Department of Occupational Therapy, Human Nutrition and Dietetics School of Health & Life Sciences Glasgow Caledonian University Glasgow United Kingdom

**Keywords:** chronic pain, digital health, digital tool, digital health intervention, mobile health, mHealth, eHealth, self-management, pain management, person-centered, patient experiences, systematic review, thematic synthesis

## Abstract

**Background:**

Research regarding the effectiveness of digital health interventions (DHIs) for people living with chronic pain is widely documented, although it is often measured against changes in clinical outcomes. To gain a comprehensive understanding of the full impact of DHIs, it is vital to understand the experience of individuals who are using them. An exploration of qualitative data regarding the experience of people living with chronic pain engaging with DHIs could provide a more in-depth account of how individuals interact and engage with such tools, uncovering the overall impact DHIs can have on the lives of people living with chronic pain.

**Objective:**

This qualitative systematic review and thematic synthesis aimed to appraise and synthesize relevant qualitative evidence on patients’ experiences of engaging with DHIs for the self-management of chronic pain symptoms.

**Methods:**

A systematic literature search of qualitative and mixed methods studies published between 2013 and 2023 was conducted across 6 databases: MEDLINE, PubMed, Embase, CINAHL, PsycINFO, and Scopus. Eligible studies included adult patients aged ≥18 years with a chronic pain diagnosis (ie, >12 weeks) reporting on the experience of engaging in a DHI for the self-management of chronic pain. The Critical Appraisal Skills Program checklist for qualitative research was used to appraise each study. Following a 3-step inductive thematic synthesis approach, the researcher performed line-by-line coding of each eligible article to identify descriptive themes. Through iterative evaluation of the descriptive themes, analytical themes that facilitated a deeper understanding of the data were derived.

**Results:**

In total, 37 qualitative and mixed methods studies were included in the review. Thematic synthesis revealed three overarching themes encompassing five subthemes: (1) personal growth, with 2 subthemes (*gaining new insights* and *renewed mindset*); (2) active involvement, with 3 subthemes (*motivation*, *improved access*, and *health care decision-making*); and (3) connectedness and support.

**Conclusions:**

A positive experience with DHIs among people living with chronic pain is achieved through an improved understanding of their condition, greater self-awareness of how symptoms impact their lives, and an increase in motivation to play an active role in their health care. DHIs promote the confidence and independence of people living with chronic pain, as well as facilitate a sense of ongoing support between routine appointments. However, DHIs may disempower people living with chronic pain by placing too much focus on their pain and should be used as an adjunct to existing care as opposed to replacing in-person appointments. To appropriately meet the needs of people living with chronic pain, the content and features of DHIs should be personalized. Development of future DHIs should use a co-design approach involving key stakeholders to ensure the needs of people living with chronic pain are met.

**Trial Registration:**

PROSPERO CRD42023445100; https://www.crd.york.ac.uk/PROSPERO/view/CRD42023445100

**International Registered Report Identifier (IRRID):**

RR2-10.2196/52469

## Introduction

### Background

Chronic pain, defined as pain that persists past the normal healing time of 3 months [1], is one of the leading causes of disability worldwide [2]. The intricate and diverse nature of chronic pain makes it a complex condition to fully understand, as pain can occur without a clear underlying cause and cannot be attributed to injury or disease. This is described as *chronic primary pain* and includes diagnoses such as fibromyalgia, chronic primary musculoskeletal pain, and chronic primary neuropathic pain. Pain that occurs as the result of an initial injury or underlying illness is known as *chronic secondary pain*, and this includes conditions such as osteoarthritis, rheumatoid arthritis, and cancer related chronic pain [3]. A systematic review assessing prevalence in the United Kingdom estimated that approximately 43.5% of the population experience chronic pain, with 12% of people living with chronic pain reporting pain that is moderately to severely disabling [4].

### Treatment

It is well known that chronic pain is multifaceted, and this can present a substantial burden to the lives of those affected [5]. Physical symptoms are often accompanied by distressing psychological, social, and economic factors that can impact every facet of an individual’s life [2]. To reflect its multifaceted nature, it is crucial that treatment addresses the diverse biopsychosocial aspects of chronic pain. Evidence indicates that treatment that uses a combination of approaches is the most effective for improving pain outcomes [6,7]; however, access to adequate multidisciplinary treatment is limited [8]. Lengthy waiting times for treatment are reported around the world [9-12], leaving patients with no other option than to rely on their general practitioner for advice [8]. Self-management, defined as “the individual’s ability to manage the symptoms, treatment, physical, psychosocial consequences, and lifestyle changes inherent in living with a long-term disorder” [13], poses a possible solution to the issue of limited access to treatment. Self-management techniques encompass a range of disciplines, integrating approaches such as tracking symptoms, engaging in exercise, relaxation techniques, and cognitive behavioral therapy (CBT) to address chronic pain symptoms [14]. Such strategies enable individuals to play an active role in their health care by treating and managing their pain on a day-to-day basis without relying on input from health services or health care professionals (HCPs). A growing body of evidence supports that self-management interventions are effective in assisting people living with chronic pain manage their symptoms [14-17].

### Digital Health

A promising mode of delivering self-management interventions is through the use of digital health technologies. The need for digitization of health care delivery became evident following the COVID-19 pandemic, as digital technologies offer an accessible, affordable, and flexible approach to managing chronic pain symptoms [18]. Digital health interventions (DHIs) use various modes of delivery, including smartphone apps, websites, wearable activity trackers, and virtual reality [19], and the literature highlights promising effects of DHIs on clinical outcomes. Pfeifer et al [20] evaluated the efficacy of app-based interventions on a variety of chronic pain conditions. An analysis of 22 studies found that apps are significantly more effective in reducing pain compared to control groups. Another systematic review that analyzed the impact of web-based and mobile-based self-management interventions on patients with chronic low back pain indicated that there was a clinically important effect both at immediate and short-term follow-up on pain relief and improved disability [21]. Furthermore, Moreno-Ligero et al [22] reviewed 22 randomized controlled trials to evaluate the effectiveness of mobile health interventions on a range of chronic pain conditions. The results suggested that the use of mobile health interventions can have beneficial effects on pain intensity, functional disability, and quality of life (QoL).

Despite an abundance of the literature outlining the benefits of DHIs for people living with chronic pain, the findings primarily focus on clinical outcomes. Research regarding clinical outcomes plays a vital role in supporting evidence-based practice, although these studies are not able to provide a comprehensive understanding of how users interact and engage with DHIs. The literature shows clear evidence that DHIs are effective in improving symptoms of chronic pain, although the full potential of DHIs is limited as engagement and retention rates within these interventions are low. A review evaluating the efficacy of web-based CBT interventions for chronic pain found that as many as 26.6% of users dropped out before completing the intervention [23]. Similarly, a more recent review exploring the DHIs for the self-management of osteoarthritis found that 6 out of the 7 studies evaluated reported less than 70% adherence to the intervention [24]. For people living with chronic pain to obtain maximum results from self-management DHIs, users need to engage with and continue to use these tools. This strongly depends on a user’s intention to use the tool [25], although the intentions behind why a user engages are poorly understood. Research regarding the barriers and facilitators to the use of DHIs among people living with chronic pain reported that too much information, a lack of tailored or personalized content, and a lack of motivation negatively affect engagement [26,27].

To overcome these barriers, it is vital that DHIs meet the actual needs of the people who are using them. To do this, developers of such interventions require a comprehensive understanding of how users experience DHIs as this could unveil aspects that will encourage engagement and retention. A qualitative exploration of how people living with chronic pain experience the use of DHIs could provide insights into how users benefit from DHIs as well as highlight the associated challenges. These findings could inform the design and development of future DHIs, resulting in more effective tools that meet the needs of people living with chronic pain.

A recent review exploring experiences of patients with chronic pain with DHIs has found that by engaging with DHIs, participants learned new skills as to how to manage their pain and gained increased confidence and reassurance with regards to managing their condition. In addition, the review stated that users were able to improve the perception of their condition, thereby obtaining a more optimistic outlook on their life [28]. The findings from this review highlighted some key recommendations for future DHIs, including providing tailored content, structured goal setting facilitated by prompts, and prioritizing features that change patients’ attitudes and behaviors. This review aims to build on this evidence with a particular focus on self-management interventions. It will also include qualitative data from mixed methods studies, which dominate this domain of research and could therefore offer valuable insights concerning the patient experience of engaging with DHIs that may have been previously overlooked.

By exploring qualitative evidence regarding patients’ experiences of engaging with specifically self-management DHIs, the findings from this review could improve the development of future DHIs by providing an in-depth explanation of how self-management DHIs impact the lives of people living with chronic pain, and which features should be included or omitted in future DHIs to enhance engagement and retention. In turn, this will uncover the full potential of how self-management DHIs can benefit people living with chronic pain.

### Aim and Objectives

The aim of this qualitative systematic review and thematic synthesis is to identify, appraise, and synthesize relevant qualitative evidence on patients’ experiences of engaging with DHIs for the self-management of chronic pain symptoms.

The objectives of this synthesis are as follows:

1. To explore how people living with chronic pain experience DHIs for the self-management of chronic pain
2. To identify factors that could improve the design and development of future DHIs

## Methods

### Overview

This systematic review and thematic synthesis was conducted and reported in accordance with the Enhancing Transparency in Reporting the Synthesis of Qualitative Research (ENTREQ) guidelines [29]; a completed checklist is presented in Multimedia Appendix 1. The protocol has been published [30] and is registered on PROSPERO (CRD42023445100).

### Search Strategy

The search strategy was adapted and applied to 6 electronic databases as follows: Embase, MEDLINE, PubMed, CINAHL, PsycINFO, and Scopus. The full search strategy for each database can be found in Multimedia Appendix 2. To retrieve relevant articles not identified on the electronic databases, forward and backward citation searches were performed on the included studies. The sample, phenomenon of interest, design, evaluation, and research (SPIDER) framework [31], which describes the sample (S), phenomenon of interest (PI), design (D), evaluation (E), and research type (R), was used to structure the search terms and develop a comprehensive search strategy relevant to the research question. The search terms that comply with each of the key characteristics of the SPIDER framework are outlined in Table 1 [31].

Each electronic database was searched on July 4, 2023, and updated on January 29, 2024. Each search was restricted to papers published in the English language; gray literature, other reviews, protocols, and unpublished studies were excluded from the review. Due to major advancements in technology, only articles published in the last 10 years (ie, 2013-2023) were included, those published before 2013 were excluded due to the potential risk of no longer being relevant. Search results were imported to EndNote (version 20; Clarivate), and duplicates were removed.

**Table 1:** Search terms for each electronic database.

Categories	Search terms
Sample	“chronic pain” OR “chronic pain” OR “persistent pain” OR “long-term pain” OR fibromyalgia OR “rheumatoid arthritis” OR osteoarthritis OR “neuro* pain” OR “musculoskeletal pain*” OR “orofacial pain” OR “visceral pain” OR endometriosis OR headache OR “irritable bowel syndrome” OR “back pain*” OR “low* back pain” OR “neck pain” OR “joint pain*” OR sciatica OR “cancer pain” OR “post surg* pain” OR “post trauma* pain” OR “complex regional pain” OR “chronic primary headache” OR “chronic primary visceral pain” OR “chronic musculoskeletal pain”
Phenomenon of interest	“telehealth” OR “digital health” OR mhealth OR ehealth OR telenursing OR telerehabilitation OR “digital intervention” OR “remote consultation” OR “electronic health” OR “internet health” OR “digital health” OR “health technolog*“ OR ”health management app*“ OR ”digital health programme“ OR ”digital health app*“
Design	”focus group*“ OR interview*
Evaluation	experience* OR perspective* OR perception* OR accept* OR satisf* OR view* OR attitude*
Research type	qualitative OR ”mixed method*“ OR “grounded theory” OR ethnography OR “thematic analysis”

### Eligibility Criteria

The inclusion and exclusion criteria were based on the key characteristics of the SPIDER framework outlined earlier [31]. Textbox 1 describes the inclusion and exclusion criteria, and a comprehensive account of the inclusion and exclusion criteria is detailed in the study protocol [30].

**Inclusion and exclusion criteria based on the sample, phenomenon of interest, design, evaluation, and research (SPIDER) framework.**

**Inclusion criteria**

- Articles reporting on adults (ie, aged ≥18 years) who were diagnosed with any chronic pain condition exceeding 12 weeks
- Articles reporting qualitative data, including mixed methods studies with a qualitative component, on patients’ experiences of engaging with a digital health intervention (DHI) for the self-management of chronic pain
- Articles reporting on DHIs, regardless of modality, to deliver self-management strategies for managing chronic pain symptoms
- Articles published from January 1, 2013, to December 31, 2023
- Articles published in the English language

**Exclusion criteria**

- Articles reporting on participants aged <18 years who have not received a diagnosis exceeding 12 weeks
- Gray literature, reviews, protocols, dissertations, and articles reporting on quantitative data from mixed methods studies
- Articles describing participant expectations and perceptions rather than the experience of engaging in a DHI
- Articles describing the experience of carers or health care professionals
- Articles describing DHIs that deliver management strategies that involve regular live health care professional contact (eg, through videoconferencing or Skype)
- Articles published before January 1, 2013
- Articles published in languages other than the English language

### Screening and Data Extraction

Articles retrieved from the electronic database were uploaded to EndNote and duplicates were removed. Articles were then imported to Rayyan (Rayyan Systems, Inc), a software that facilitates screening in the systematic review process. After thoroughly discussing the eligibility criteria to ensure that reviewers had a comprehensive understanding of which studies to include and exclude, all of the titles and abstracts were screened by 2 independent reviewers (AM and HM). There was a high rate of agreement, as 89% (33/37) of the papers were agreed upon. Where disagreement between reviewers did occur, this was resolved through discussion between 2 reviewers through a thorough investigation of each paper against the inclusion and exclusion criteria (AM and MI). In the following phase, full-text articles of the included titles and abstracts were obtained, and the same 2 reviewers (AM and HM) assessed the full-text articles for eligibility. During this round, there was a 96% agreement rate, and again, the reviewers resolved any disagreement through discussion. In one case, resolution between the reviewers could not be reached and a third reviewer was consulted (FM).

A data extraction table was devised by the research team, and 2 reviewers (AM and HM) independently extracted data from 20% (7/37) of the included articles. Data extraction forms were then compared between reviewers to ensure that the key characteristics of each study were extracted in a systematic and standardized way. The first author (AM) then continued to extract data from the remaining included articles.

### Quality Assessment and Critical Appraisal

Due to the subjective nature of qualitative research, assessing the methodological quality of included studies is a crucial step to ensure the validity of results. The Critical Appraisal Skills Program (CASP) qualitative research checklist was chosen to appraise each article as the checklist is endorsed by Cochrane’s Supplemental Guidance for Inclusion of Qualitative Research [32]. Assessment consists of scoring 10 questions regarding methodological quality as “yes,” “can’t tell,” or “no.” The questions concerned issues such as research design, recruitment strategy, data collection, ethics, data analysis, and presentation and interpretation of findings.

Two independent reviewers (AM and HM) assessed the CASP checklist criteria before commencing the quality assessment phase. This was to fully understand the criteria before applying each paper against the checklist. The 2 reviewers appraised 20% (7/37) of the included articles. After comparing scores of the subsample, which reached a high degree of agreement (6/7, 85%), discrepancies were resolved through discussion. The first author then continued to appraise the remaining 30 articles. There are currently no standardized rules for the exclusion of studies based on their appraisal score; therefore, all studies were included in the synthesis. The average quality appraisal score of the included articles was high (ie, 8.8/10).

### Thematic Synthesis

A 3-step inductive thematic synthesis approach as described by Thomas and Harden [33] was applied to the results and discussion sections of each included article. This methodology was selected as it is recommended by the *Cochrane Handbook for Systematic Reviews of Interventions* [32]. The 3-step methodology provides an opportunity to extend beyond the findings of the primary studies by deriving analytical themes from descriptive themes, which in turn facilitates a deeper conceptual understanding of the data. Qualitative evidence syntheses have faced criticism for decontextualizing the results of individual studies [33]. Therefore, before the 3-step thematic synthesis approach began, each paper was read in its entirety to grasp a full understanding.

In step 1, the first author (AM) imported the primary studies to NVivo (Lumivero), where line-by-line coding of the results and discussion sections of each primary study was performed to identify concepts within the data. In step 2, items coded in step 1 were grouped together to create descriptive themes, closely aligning with the themes identified in the primary research. Finally in stage 3, by continually examining and interpreting the descriptive themes, analytical themes that reflected a deeper understanding of the data were derived.

## Results

### Study Selection

**Figure 1:**
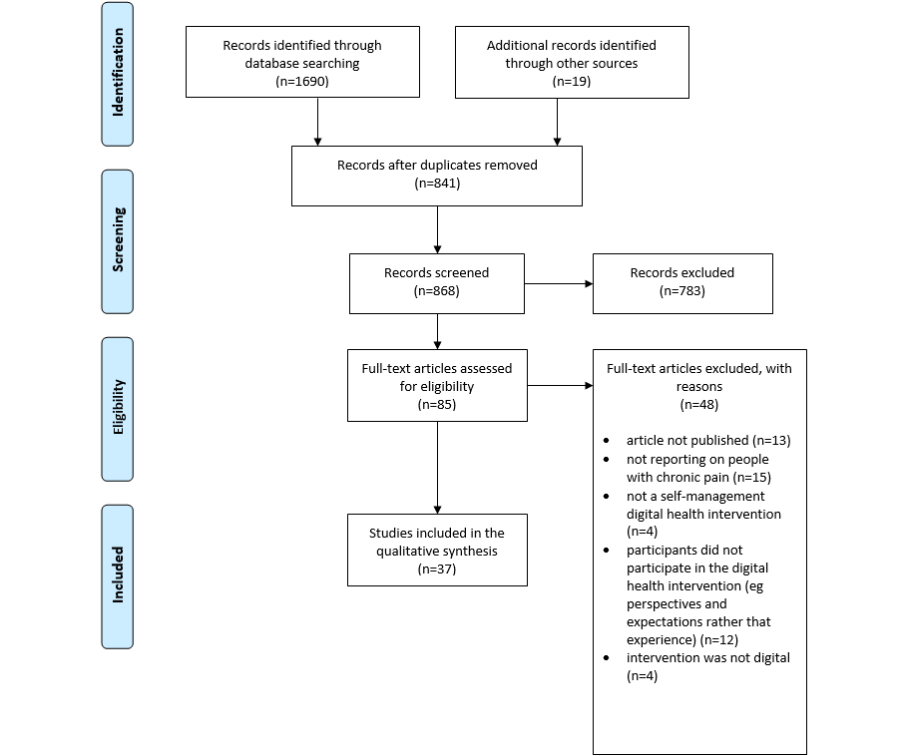
PRISMA (Preferred Reporting Items for Systematic Reviews and Meta-Analyses) study selection flowchart. DHI: digital health intervention.

The search yielded 1709 records. After duplicates were removed, the titles and abstracts of 868 (51%) records were screened, and 85 (5%) records were retrieved for full-text screening. A total of 48 (57%) records were excluded; reasons for exclusion can be found in the PRISMA (Preferred Reporting Items for Systematic Reviews and Meta-Analyses) flowchart (Figure 1; Multimedia Appendix 3). A total of 37 (43%) records met all eligibility criteria and were included in the review (Multimedia Appendix 4 [34-70]).

### Study Characteristics

The 37 included studies used a range of qualitative methods to collect data, including interviews [34-62], focus groups [63-67], questionnaires [68], and a combination of interviews and questionnaires [69,70]. In total, 18 (49%) studies conducted interviews remotely [34,38,41-43,45-49,52,54-56,58,62,69,70] and 5 (14%) studies had the option of both in-person or remote interviews [37,39,53,59,61]. A total of 2 (5%) studies did not detail how the interviews took place [36,60]. Focus groups were conducted in person in 2 (5%) studies [63,64], on the web in 2 (5%) studies [65,66], and 1 (3%) study used a combination of both in-person and online methods [67].

The sample sizes ranged from 7 to 36, with a total of 707 participants. A total of 4 of 37 (11%) studies included only female participants [37,45,59,65], and the remaining 33 (89%) studies included both male and female participants [34-36,38-44,46,48-58,60-64,66-70]. The interventions were investigated in the United States [42,44,45,51,54,60,61], the Netherlands [35,46,59,62,66,69,70], Canada [40,41,53,67], the United Kingdom [34,36,37,58], Australia [43,47,49], Sweden [39,50,55,63], Germany [48,52], New Zealand [64], France [68], Belgium [65], Norway [38], and Turkey [57], and 1 study was conducted in both Sweden and Denmark [56].

In terms of chronic pain conditions, 6 of 37 (16%) studies did not differentiate between chronic pain diagnoses [38,40,42,44,47,55], while the remaining 31 (84%) studies focused on specific chronic pain conditions: knee or hip osteoarthritis [34,39,41,43,49,53,54,63,64], rheumatoid arthritis [36,48,57,62,69,70], low back pain [35,52,56,61,68], cancer pain [45,51,65,67], musculoskeletal pain [46,50,60], chronic pelvic pain [37], irritable bowel syndrome [58], and temporomandibular disorder [59], and 1 study evaluated rheumatoid arthritis, psoriatic arthritis, and ankylosing spondylitis [66].

A range of digital tools were used to deliver the telehealth interventions; smartphone apps [35-38,42,44,48,53, 56,57,62,64,66,68,70], web-based interventions [34,39,41,43,45,46,49-52,54,58-61,65,69], virtual reality headsets [40,67], social media [47], and wearable activity trackers [63], and 1 intervention offered both a smartphone app and web-based platform [55]. The purpose of the interventions varied, with most studies using a multitude of different intervention components [34,39,41-43,45,46,50, 51,53,55,56,59,62,65,69]. While the remaining interventions also incorporated a range of components, the main focus of the intervention was on CBT or mindfulness and meditation strategies [37,38,40,58,67], self-monitoring and symptom tracking [36,48,60,64,66,70], physical activity [35,49,57,63,68], virtual auricular point acupressure [44,61], social support [47], and shared decision-making [52,54].

The study characteristics pertaining to each included article can be found in Multimedia Appendix 4, and a table of intervention characteristics can be found in Multimedia Appendix 5 [34-70].

### Quality Assessment

#### Overview

Of the 37 studies, 35 (95%) studies included in the review were rated as “high” quality by scoring >7 out of 10 [34-46,48-68,70]. A total of 2 (5%) studies were rated as “low” quality [47,69], failing to describe multiple key aspects, such as the research design, recruitment strategy, data collection, and data analysis process; 2 (5%) studies did not clearly state the aims of the research [44,51]; 4 (11%) studies did not provide thorough detail as to how the data were collected [36,47,60,69]; and 3 (8%) studies failed to give an in-depth description of the data analysis process [47,69,70]. The item that was scored most poorly throughout the included studies was the consideration of researcher bias, with 26 (70%) studies lacking sufficient detail on the relationship between the researcher and the participants [34-37,40-43,46-48,51,53-57,60-62,65-70]. The full quality appraisal for each study can be found in Multimedia Appendix 6.

#### Thematic Synthesis Findings

The data revealed 3 analytical themes that encapsulated patients’ experiences of DHIs for chronic pain management as follows: (1) personal growth, with 2 subthemes (*gaining new insights* and *renewed mindset*); (2) active involvement with 3 subthemes *motivation*, *improved access*, and *health care decision-making*; and (3) connectedness and support. The subsequent sections provide a description of each theme.

##### Theme 1: Personal Growth (“I Could See the Change That Was Happening”)

###### Overview

A dominant theme throughout the studies was that participants experienced personal changes that made them grow and evolve as individuals, facilitating personal growth both physically and emotionally (34/37, 92%). Improved understanding of their condition led participants to adopt greater self-awareness of their symptoms. Participants obtained an optimistic outlook on life through enhanced confidence and independence, ultimately accomplishing a sense of acceptance of their diagnosis. This analytical theme encompasses 2 subthemes: gaining new insights and a renewed mindset.

###### Gaining New Insights: “I Understand How My Brain Controls the Pain”

Most studies reported that self-management DHIs improved participants’ self-awareness, facilitating a deeper understanding of their condition [34]. Participants were able to recognize patterns and triggers that exacerbate pain by tracking their symptoms within web-based and app-based interventions [36,54,58,64]: “The one thing it did make me do was look at my diet more and made me associate different reactions and triggers for my IBS” [58]. This sense of enhanced self-awareness allowed participants to understand the relationship between different variables and how this impacted their pain, enabling them to make practical changes to their lifestyles resulting in better symptom management.

Participants were able to identify thoughts and emotions that had a negative impact on their pain using CBT [58]. Mindfulness and meditation sessions equipped participants with various skills to reduce stress and anxiety [38]. Some participants were unaware that CBT could be useful for pain management before participating in the intervention [50]. Many of the interventions encouraged participants to self-reflect, which enhanced their overall understanding of the different aspects of their pain [38,43,46,50,58,60,64].

Interventions that supported physical activity gave patients insight into what exercises they can do to alleviate pain [41,43,50,56,57,62,63]. This made participants aware of how their overall health can improve:

Because I’m doing a bit of exercise in combination with the diet. So, I can see if I continue this part, not only my knee will benefit, [but] also my health.

41

Conversely, some studies found that enhanced insights into pain can also have a negative effect [37,38,40,46,62,67,70]. Participants occasionally found that too much focus on their symptoms made them aware of how poorly they were coping, “Every time I got those registrations...you have to start to notice, and feel, that you are not actually doing so well after all” [38]. For some, this increased awareness was perceived as “heavy to encounter” [46], particularly for patients who are trying to forget about their pain. It was also highlighted that regularly tracking and monitoring symptoms can risk placing too much emphasis on the negative aspects of an individual’s health [55,60,62]: “It just triggered me to start looking at my overall health” [60]. One study reported that some elements of CBT were “too confronting for patients” [62], and another study that delivered a virtual reality computer-simulated environment through a binocular headset found that focusing on pain resulted in participants experiencing more pain [67].

###### Renewed Mindset: “It’s Brought a Positive Into My Lifestyle”

The studies reviewed suggested that DHIs instilled confidence and reassurance in many of the participants. Information within the DHIs validated some participants’ beliefs and reassured them that they were not alone [58]. Collecting data on pain and other symptoms increased participants’ confidence when it came to making decisions about treatment [54]. Participants felt reassured that self-management strategies were effective based on data collected [35], particularly with the help of graphical summaries that displayed patterns of their pain [48]:

I felt that my back was very painful this week, but actually my pain score after doing the exercises is decreasing. That is, for me, a reminder I’m going in the right direction, and I find that very reassuring.

35

Video instructions incorporated into exercise-based interventions promoted a sense of confidence by reassuring participants that they were completing the exercises correctly [59]. One study noted that exercises for managing knee pain promoted participants confidence to maintain their lifestyle and not give up because of their pain: “It’s helped me not only with my mobility but my self-confidence to be able to go, yeah, I can get up there all right and come down there” [49].

Participants developed an optimistic attitude about their pain throughout CBT sessions [38,43,58], which offered participants alternative coping strategies so that they were confident enough to not depend heavily on medication: “I got to the stage where I wouldn’t leave the house without making sure the medication was in my handbag...while I was doing the trial I never thought like that at all” [58]. Another article reported that app-based CBT was effective in changing participants’ perspectives by providing hope that they can live a good QoL despite their pain [38]. In one study, family members noticed an increase in independence because of web-based pain-coping skills training [51]. Educational sessions were also found to be effective in promoting acceptance in some participants by changing the way they think about their pain [46,56]. Some studies reported that participants could use the DHIs to self-manage their symptoms instead of relying on input from HCPs [52,56,65]: “When you understand everything in the program and know how to apply this information into daily life, a conversation with a health care provider wouldn’t be necessary anymore” [65].

By changing their perceptions of pain, many participants achieved a degree of acceptance which resulted in them coming to terms with their diagnosis [38,43,46,51,52,56,69]: “I’m just trying to learn to live with it [OA], and I know it’s a cliché, but just accept the new normal, and not be living in the past and remembering how when I could just walk all day and not worry about it” [43].

On the other hand, some studies noted the adverse experiences of engaging in the DHIs on participants’ psychological well-being [37,38,40,48,49,63,65,67]. Monitoring disease activity on a smartphone app evoked negative thoughts for one participant:

If you dwell too often on your own disease activity, it can be associated with negative thoughts. You may be more likely to get into such a negative vortex.

48

In an intervention that used a virtual reality immersive experience, some participants experienced anxiety, depression, and stress that aggravated their pain [67].

###### Theme 2: Active Involvement (“I Would Never Have Done It If It Hadn’t Been Digital”)

####### Overview

This theme illustrates how DHIs can promote patients to play an active role in the management of their chronic pain. As opposed to being a “passive” recipient of care, accessible self-management resources motivated participants to act and engage in conversations about their health care. This analytical theme is underpinned by 3 descriptive themes (motivation, improved access, and health care decision-making).

####### Motivation: “I Dare Do More Things!”

The studies continually described how participants were motivated to engage in behaviors that positively impacted their pain, and therefore promoted a greater sense of control [39,43,44,65,68]: “Thanks to the program, I got the feeling that I had more control on my pain” [65]. Participants expressed a sense of motivation that went beyond the goal of the intervention, such as inspiring them to lose weight [49] or to change their occupation to something that was less physically demanding and better suited to their needs [43]. The results from the exercised-based programs suggested that participants were motivated to exercise more as a direct result of the DHI [35,41,52,56-58,62,63,69]: “Thanks to the app, I could see what exactly it was I was supposed to do...That definitely increased how often I exercised” [35].

Notifications either as alerts or emails were noted to have a motivating effect on participant engagement throughout the interventions [34,35,39,41,43,49,57,59,63]: “Reminders and notifications are my favorite, I love that some notifications motivate me by saying ‘how about moving? have a nice day!’” [57]. Nevertheless, some of the studies found that notifications and prompts had a negative impact on participants feeling of empowerment [49,63]. In a study that used wearable activity trackers to encourage physical activity, notifications prompting participants to reach their step goal were perceived as distressing when participants were in too much pain to meet their target [63].

Although most participants experienced enhanced motivation, some advised that they felt less motivated to engage with the DHI [39,41,45,49,58,62,63]. Participants struggled to stay motivated when completing a home-based yoga program due to outside distractions and other commitments [45]. Once pain had improved, some participants did not have the same motivation to continue with the program: “The pain was generally reduced...and then I did not feel as motivated as before so I quit doing the exercises” [39]. When participants felt demotivated and had not completed the recommended exercises, SMS text messaging reminders evoked feelings of shame or guilt in one study [49], and another described how the weekly content delivered to participants through a web-based self-care intervention felt like “unfinished homework” [41].

####### Improved Access: “It’s Awfully Good to Have the Tools at Home”

Participants were motivated to play an active role in their health care because of convenient and flexible access to self-management resources. Participants in the app-based interventions commented on how having quick, convenient, and flexible access to self-management strategies encouraged them to take action [35,38,59,64]:

You’ve got your phone with you, and you can just [get it] ...done. Whereas, with a piece of paper, you’ve got to go and sit down somewhere, and you’ve got to write it out.

64

The flexibility of being able to choose when and where to engage in the various self-management techniques enabled participants to take control over their symptoms at a time that suited them, stimulating engagement with the DHIs [38,39,50,51,59,65]: “It’s awfully good to have the tools at home to use when you can ﬁnd the time” [51]. Participants advised how they were able to access the self-management strategies while on a waiting list for specialized treatment:

Yes, it was also like, there was such a long waiting list for primary care. So I thought...this...this I can start doing right away.

39

The affordability of the interventions was also valued, as participants who might not be able to afford a gym membership now have access to a range of exercises to relieve pain [41].

####### Health Care Decision-Making: “This Made Me Feel Safe to Share Those Thoughts”

Various features of the DHIs improved patient-physician communication during consultations, enabling them to play an active role in making decisions about their health care [35,36,41,46,48,50,52,54,60,63,66,70]. In a web-based intervention, participants noted how the CBT modules empowered them to become more involved in their rehabilitation as they had the tools to have meaningful discussions with their HCP [50]. Symptom tracking proved valuable in accurately portraying trends, highlighting exactly when pain improved or declined, which may provide insights that may have otherwise been overlooked [36,48,54]: “I don’t think in that half hour that you’ve got that consultation doesn’t always show the bigger picture of what you’re actually dealing with” [36]. Some participants even advised their HCP that they wanted to change their treatment plan because of the intervention: “I said I didn’t want the knee replacement yet because I found these exercises—I told him I was doing these exercises and I thought, ‘they feel great.’ I can walk more. I can do quite a few different things” [41].

####### Theme 3: Connectedness and Support (an “In-the-Pocket Friend”)

This theme articulates how DHIs are valued as a source of ongoing support outside of routine health care appointments, with some participants even describing the intervention as a “friend when in need” [38]. The DHIs were found to be useful in fostering a sense of ongoing support once treatment had ended [55,65]. Others found the interventions helpful in between consultations as they could rely on the digital tool for assistance:

After listening to the therapist, I would come home and still have questions or forgot what the therapist said. Then, I had something to fall back on, and that was very pleasant.

35

Of the 37 studies, 6 (16%) studies described how the digital format of the intervention made it feel as though they had a digital “friend” or friendly motivating supporter [38,39,41,47,56,65]. In an app-based pain management CBT intervention, participants indicated that they perceived the app as a potential “friend when in need” that could offer social support to help participants feel less lonely: “If you are in a lot of pain, then you are...quite alone...I have felt that you can withdraw and listen to the soothing voice...it has given me a break..., and it has provided relaxation” [38]. Participants appreciated the conversational style of an online pain management program [65]. Another study described the app format as “an in-the-pocket friend” that encouraged participants to keep going:

Yes, a bit like an exercise partner who asks ‘hey, shouldn’t we go work out today?’ and then you actually might get going. Where on the other hand, if you were on your own, you might forget about it or take the easy way out.

56

Sharing experiences with others was described as “emotionally cathartic” [47] in a social media–based intervention which encouraged participants to interact on various social media platforms.

However, several participants wished to be able to interact with others throughout the DHI, but the intervention lacked the features to do so [62]. Many studies noted how the DHIs lacked HCP communication [39,41,49,56,58,60,62,63,66]. Some participants preferred being able to interact with their HCP as opposed to a virtual agent:

For me, this [program] couldn’t replace a session with a therapist. Despite that the program was very valuable, I prefer a face-to-face conversation with a HCP, where you can interact with a real person.

65

Despite providing a source of ongoing support and social connectedness for many participants, it was highlighted that DHIs should be used as an adjunct to usual care and should not replace routine face-to-face appointments [35,37,42,52,56,60,63,65,66]: “The app is good progress, but it’s not yet a replacement of the physical therapist” [35].

A list of studies adhering to each subtheme can be found in Tables 2-4. Multimedia Appendix 7 provides additional participant quotations from the studies illustrating each theme.

**Table 2:** Themes identified in each study: personal growth.

Studies including theme 1 (personal growth)	Presence of the subthemes	
	Gaining new insights	Renewed mindset
Algeo et al [34], 2017	✓^a^	
Arensman et al [35], 2022		✓
Austin et al [36], 2020	✓	✓
Ball et al [37], 2020	✓	✓
Bostrom et al [38], 2022	✓	✓
Cronstrom et al [39], 2019	✓	
De Groef et al [65], 2023	✓	✓
Garrett et al [40], 2017	✓	
Garrett et al [67], 2020	✓	✓
Godziuk et al [41], 2023	✓	✓
Grolier et al [68], 2023		
Hogan et al [42], 2022	✓	
Hoving et al [69], 2014	✓	✓
Jeon et al [43], 2019	✓	✓
Kawi et al [44], 2022		
Knoerl et al [45], 2022		
Lamper et al [46], 2021	✓	✓
Merolli et al [47], 2016	✓	
Muehlensiepen et al [48], 2023	✓	✓
Nelligan et al [49], 2020	✓	✓
Nordin et al [50], 2017	✓	✓
Östlind et al [63], 2022	✓	✓
Overton et al [64], 2023	✓	
Rini et al [51], 2018	✓	✓
Schlett et al [52], 2022	✓	✓
Seppen et al [70], 2020	✓	✓
Seppen et al [66], 2023	✓	
Shewchuk et al [53], 2021	✓	
Stern et al [54], 2022	✓	✓
Svanholm et al [55], 2023	✓	✓
Svendsen et al [56], 2022	✓	✓
Tonga et al [57], 2021	✓	
Tonkin-Crine et al [58], 2013	✓	✓
van der Meer et al [59], 2022	✓	✓
Whitney et al [60], 2018	✓	✓
Yeh et al [61], 2022	✓	
Zuidema et al [62], 2019	✓	✓

^a^Subtheme was identified within the article.

**Table 3:** Themes identified in each study: active involvement.

Studies including theme 2 (active involvement)	Presence of the subthemes		
	Improved access	Motivation	Health care decision-making
Algeo et al [34], 2017		✓^a^	
Arensman et al [35], 2022	✓	✓	✓
Austin et al [36], 2020	✓		✓
Ball et al [37], 2020		✓	
Bostrom et al [38], 2022	✓	✓	
Cronstrom et al [39], 2019	✓	✓	
De Groef et al [65], 2023	✓	✓	
Garrett et al [40], 2017	✓		
Garrett et al [67], 2020			
Godziuk et al [41], 2023	✓	✓	✓
Grolier et al [68], 2023		✓	
Hogan et al [42], 2022			
Hoving et al [69], 2014		✓	
Jeon et al [43], 2019		✓	
Kawi et al [44], 2022	✓	✓	
Knoerl et al [45], 2022	✓	✓	
Lamper et al [46], 2021		✓	✓
Merolli et al [47], 2016	✓	✓	
Muehlensiepen et al [48], 2023		✓	✓
Nelligan et al [49], 2020	✓	✓	
Nordin et al [50], 2017	✓	✓	✓
Östlind et al [63], 2022		✓	✓
Overton et al [64], 2023	✓		
Rini et al [51], 2018	✓	✓	
Schlett et al [52], 2022	✓	✓	✓
Seppen et al [70], 2020	✓	✓	✓
Seppen et al [66], 2023	✓	✓	✓
Shewchuk et al [53], 2021		✓	✓
Stern et al [54], 2022		✓	✓
Svanholm et al [55], 2023		✓	✓
Svendsen et al [56], 2022	✓	✓	
Tonga et al [57], 2021	✓	✓	
Tonkin-Crine et al [58], 2013		✓	
van der Meer et al [59], 2022	✓	✓	
Whitney et al [60], 2018		✓	✓
Yeh et al [61], 2022			
Zuidema et al [62], 2019		✓	

^a^Subtheme was identified within the article.

**Table 4:** Themes identified in each study: connectedness and support.

Studies including theme 3 (connectedness and support)	Presence of theme
Algeo et al [34], 2017	
Arensman et al [35], 2022	✓^a^
Austin et al [36], 2020	
Ball et al [37], 2020	✓
Bostrom et al [38], 2022	✓
Cronstrom et al [39], 2019	✓
De Groef et al [65], 2023	✓
Garrett et al [40], 2017	
Garrett et al [67], 2020	
Godziuk et al [41], 2023	✓
Grolier et al [68], 2023	
Hogan et al [42], 2022	✓
Hoving et al [69], 2014	✓
Jeon et al [43], 2019	
Kawi et al [44], 2022	
Knoerl et al [45], 2022	✓
Lamper et al [46], 2021	✓
Merolli et al [47], 2016	✓
Muehlensiepen et al [48], 2023	✓
Nelligan et al [49], 2020	✓
Nordin et al [50], 2017	
Östlind et al [63], 2022	✓
Overton et al [64], 2023	
Rini et al [51], 2018	✓
Schlett et al [52], 2022	✓
Seppen et al [70], 2020	
Seppen et al [66], 2023	✓
Shewchuk et al [53], 2021	
Stern et al [54], 2022	✓
Svanholm et al [55], 2023	✓
Svendsen et al [56], 2022	✓
Tonga et al [57], 2021	
Tonkin-Crine et al [58], 2013	✓
van der Meer et al [59], 2022	
Whitney et al [60], 2018	✓
Yeh et al [61], 2022	
Zuidema et al [62], 2019	✓

^a^Subtheme was identified within the article.

### Summary of Synthesis

The analytical themes that have been derived from the qualitative synthesis suggest that while many patients report positive experiences of participating in DHIs, the content needs to be tailored to fit the patients’ needs otherwise DHIs can have a negative effect on patient outcomes or can lead to poor engagement. This suggests that “a one-size-fits-all” approach is not always effective in achieving the aims of patients wishing to self-manage their chronic pain symptoms.

DHIs can support the acquisition of knowledge and understanding through improved self-awareness, while encouraging independence and confidence in patients, ultimately stimulating self-efficacy. Accessibility and features that foster motivation, confidence, and promote a sense of ongoing support outside of appointments with HCPs should be prioritized in the future development of DHIs as this is likely to facilitate engagement and improve health outcomes for people living with chronic pain.

## Discussion

### Overview

This qualitative systematic review aimed to identify, appraise, and synthesize qualitative evidence on the experience of engaging in self-management DHIs among patients with a varying range of chronic pain conditions. The findings revealed that most participants reported a positive experience with DHIs through improved self-awareness, confidence, and independence; through enhanced motivation; and by playing an active role in the management of their chronic pain condition. However, contradictory perspectives exist within each theme, creating a discrepancy as to what features optimize the acceptability of DHIs. These contradictions suggest that for patients to engage with and benefit from digital self-management interventions for chronic pain, it is crucial that content is tailored to the needs of the targeted patient group. The involvement of patients throughout the design and development of DHIs for the self-management of chronic pain could ensure that these needs are met. In the subsequent sections, each theme will be discussed in relation to existing literature and will outline how the results can inform the development and delivery of future DHIs.

### Principal Findings

The first analytical theme “personal growth” relates to the personal attributes developed by participating in the DHIs. Patients became more self-aware of how pain impacted other aspects of their life, ultimately leading to a better understanding of their condition and improved pain management [41,58,60,64]. These findings echo the results of a review by Strain et al [28] that explored patients’ experience with a range of digital pain management interventions and reported that DHIs enabled people living with chronic pain to become aware of how patterns of behavior could impact their pain symptoms, creating meaningful behavior change. Another review of qualitative studies on user experience with DHIs within psychosis found similar results, suggesting that participants’ increased insight allowed them to understand links between their symptoms, mood, and behavior, which equipped them with the tools to better manage their symptoms [71]. On the other hand, many participants did not desire an enhanced level of self-awareness, coinciding with the results of an umbrella review of experiences of DHIs across a range of chronic conditions which found that too much information on a participant’s health can be a risk without professional interpretation [72]. This suggests that although features that facilitate self-awareness such as symptom tracking and CBT should be prioritized in the design of future DHIs, caution should be taken during the design phase so that DHIs do not place too much emphasis on negative aspects of health or produce summaries of self-reported symptoms that could be misinterpreted.

Further to the first analytical theme of “personal growth,” participant’s attitude about their pain improved as patients reported feeling more confident, reassured, and independent. Confidence to communicate pain with others is a crucial factor for people living with chronic pain, who commonly suffer in silence because of barriers to treatment and the associated stigma of a diagnosis [73]. Taylor et al [72] reported similar findings, stating that participants experienced feelings of confidence and reassurance from DHIs across various chronic conditions, as participants could use their logged data to legitimize their reason for contacting an HCP. Patients also became empowered to be more independent [43,51,52,55,56,58,60,65], reporting that they did not have to rely on medication [65] or advice from HCPs [58] as they were able to use self-management strategies to manage their pain. Due to the demands and challenges of living with chronic pain, it is common for patients to lose their sense of “self,” as many individuals are unable to upkeep their lifestyle and continue with activities they previously enjoyed [74]. By promoting confidence and enabling participants to become more independent, DHIs can inspire patients to have a more optimistic outlook on life, encouraging them to maintain their usual activities without fear of exacerbating their pain which will ultimately improve their QoL.

Another notable experience was that participants reported gaining a degree of acceptance regarding their diagnosis [38,43,46,51,52,56,69]. Research supports that pain acceptance and optimism are crucial steps toward self-management [75]. A systematic review of the concept of “self” among patients with chronic pain reported that negative evaluations of oneself can lead to more complications with daily functioning, and when an individual views their sense of “self” as more positive, they are more likely to experience better functioning [74]. This idea is further supported by research that states improved self-efficacy and pain acceptance are effective strategies in reducing future pain disability and protecting individuals against chronic pain [76,77]. The findings from this review suggest that patients with chronic pain can benefit from improved pain self-efficacy and acceptance of their diagnosis when engaging with self-management DHIs, and this could carry massive potential in reducing the future burden of pain.

Second, “active involvement” emerged as an analytical theme. This theme highlighted how participants were motivated to engage in positive health behaviors through increased access to self-management resources, as they were able to actively manage their pain outside of a health care setting. Strain et al [28] also reported that digital pain management interventions encouraged participants to play a more active role in their care, strengthening the idea that digital health can be an effective means of alleviating the strain on the health care system by encouraging people living with chronic pain to take control into their own hands. The importance of taking action to reduce chronic pain symptoms was demonstrated in a population-based study, which showed that patients who relied on passive coping strategies (such as pain medication and rest) had as many as 3 times the amount of health care appointments and double the level of disability compared to those who adopted active coping strategies such as exercise [78]. In particular, exercise-based interventions that encouraged participants to hit daily exercise targets were found to be motivating [35,41,52,56-58,62,63,69]. In many of the studies, notifications motivated participants to engage with the digital tool [34,35,39,41,43,49,57,59,63], a finding consistent with other reviews on patients’ experiences with DHIs [71,72]. On the other hand, notifications caused unnecessary distress for some, particularly when targets had not been reached [41,49]. By stimulating motivation to engage in health behaviors that could improve levels of pain, DHIs could potentially play a major role in improving pain-related outcomes in people living with chronic pain. However, it is important to set realistic and achievable goals, as unobtainable targets run the risk of creating an adverse effect on motivation [63]. These findings suggest that participants should be given the option to customize the frequency of notifications to align with their treatment goals, as this will avoid notifications having a negative effect and hindering engagement.

Access to specialized pain services poses a barrier to effective treatment, with many patients reporting low satisfaction with the quality of pain management care they receive because of long waiting times and lack of interest from HCPs [8]. The flexibility of being able to choose when and where to engage in the various self-management techniques motivated participants to take control of their symptoms at a time that suited them [38,39,50,51,59,65].

Another way that the participants felt that they were playing a more active role in their health care was by contributing more during consultations with HCPs. Equipping patients with tools that enable them to actively participate in health care discussions will ensure that their treatment is personalized to their specific needs, which has the potential to improve various health outcomes.

The third analytical theme “connectedness and support” outlines how DHIs were found to be useful in fostering a sense of ongoing support between health care appointments and at the end of treatment [35,55,65]. Social support is crucial in the treatment of chronic pain as research indicates that higher levels of social support are associated with lower levels of pain [79]. A systematic review exploring factors affecting engagement suggested that technology supporting human interaction with both peers and HCPs could promote engagement and enrollment as this allows users to obtain social support quickly and effectively [80]. The findings from this review demonstrate that DHIs are effective tools for facilitating social support, although social support in the included articles was perceived through time spent with family and friends and communication with virtual avatars embedded into the interventions, which many participants referred to as a virtual “friend.” Most studies included in the review lacked features that allowed participants to directly interact with other patients within the intervention. In the one study that facilitated sharing experiences with others, participants described being connected with others as “emotionally cathartic” [47], although it should be highlighted that this study scored poorly on the CASP appraisal tool; therefore, these findings should be interpreted with caution. This is in line with the findings of a review exploring social media uses for various health purposes, which reported that online communities can provide invaluable advice by offering emotional support as well as sharing information such as medication, diagnosis, treatment options, and self-care activities [81].

Chronic pain is often regarded as an “invisible illness,” with many patients having faced skepticism and suspicion of exaggeration or even accused of lying about their condition [82-85]. Therefore, it is important that patients with chronic pain obtain support from others in a similar position as only those with lived experience can truly comprehend the extent to which chronic pain interferes with daily life. Strain et al [28] reported similar findings, stating that interactions with other users or with HCPs have the potential to reduce anxiety and loneliness, although the anonymity offered through DHIs was also valued; therefore, it could be that users have the option to stay anonymous. Features enabling communication with HCPs might facilitate patient engagement and continued use as many patients reported an impersonal feel to DHIs as they were unable to receive feedback and did not experience accountability. Others mentioned a preference for being able to interact with an HCP as opposed to a virtual agent [65]. The inclusion of interactive features could stimulate the sense of ongoing support; however, DHIs should enhance traditional face-to-face care as opposed to serving as a replacement. Future DHIs could incorporate an element of interaction with HCPs that could be continued into routine appointments allowing for more effective consultations.

### Limitations

This review should be interpreted with caution, as several limitations exist. First, this synthesis only included studies from 2013 onward in an attempt to capture the most relevant and up-to-date DHI systems that have been developed over the last decade. Consequently, the findings reported in this review may not reflect the experiences of patients with chronic pain who engaged with digital self-management interventions before 2013, which could be dramatically different due to advancements in digital technology. Second, as with all qualitative syntheses, this review relies on secondary data that were analyzed through a varying range of theoretical underpinnings and methods of data analysis [86]. Another limitation of this review is that the diversity of the samples, which included a range of chronic pain conditions, made it challenging to identify which DHI would be the most beneficial for each individual patient group. Furthermore, the DHIs encompassed multiple intervention components, which were often intertwined and difficult to separate in the results. Therefore, this review could not always draw conclusions about which intervention components elicited which experiences, reducing the richness of the findings. Another limitation was that many of the studies did not report the frequency and duration of the intervention, nor how long after the intervention the qualitative data were collected. Thus, it is unknown whether the timing or frequency of the DHI influenced the patient experience. Of the 33 included studies that did detail the intervention length, only 1 (3%) study was conducted for 12 months, with the remaining studies lasting ≤6 months; therefore, the long-term experience of DHIs for the self-management of chronic pain could not be synthesized and remains not fully understood. Future trials should explore the experience of DHIs for the self-management of chronic pain over a period of 2 years to fully grasp the long-term experience of these interventions. The largest sample size was 36 participants, with 86% (32/37) of studies obtaining samples of <30 participants. It is important to consider sampling bias that may have occurred among the studies with small samples, and that the results may not be representative of all patients with chronic pain. Future studies should involve larger samples so that the results can be generalized to the larger population. Another limitation to consider is that most of the studies were conducted in high-income countries, as the implementation of DHIs in low- and middle-income countries presents several challenges, such as resource limitations and costs [87].

### Conclusions

The findings from this systematic review and thematic synthesis suggest that DHIs for the self-management of chronic pain elicit mostly positive experiences among people living with chronic pain; however, opinions on which features are optimal for improving pain outcomes are diverse, highlighting that a “one-size-fits-all” approach is not effective. Personalized content should be incorporated to ensure that DHIs address the needs of this patient group, and content should be customizable to avoid negative effects on adoption and engagement. This could be achieved by involving patients in the early stages of intervention design and development; therefore, a co-design approach for the creation of future DHIs for the self-management of chronic pain is advised. The results of this review can be used to inform the development of future DHIs, suggesting that such DHIs should be accessible, convenient, and flexible and be easily integrated into patients’ lives without exacerbating the burden on those already coping with a range of challenging symptoms. Intervention components, including CBT, symptom tracking, exercise programs, and educational content should be prioritized. These features have demonstrated improvements in patients’ self-awareness, confidence, independence, and acceptance, all while fostering motivation to engage in positive health behaviors, decisions about treatment and care with HCPs, and the feeling of being supported outside of routine health care appointments. The findings from this review point to a lack of social networking functionalities with both peers and HCPs in DHIs for the self-management of chronic pain, suggesting a potential gap in the research. Further investigation surrounding the availability of such social features should be conducted, as social networking functionalities could provide a means of support that could relieve the burden on both HCPs and services.

## Appendix

Multimedia Appendix 1 Enhancing Transparency in Reporting the Synthesis of Qualitative Research (ENTREQ) checklist.

Multimedia Appendix 2 Search strategy for each database.

Multimedia Appendix 3 PRISMA checklist.

Multimedia Appendix 4 Summary of study characteristics from each qualitative or mixed methods study included in the review (N=37).

Multimedia Appendix 5 Summary of intervention characteristics from each qualitative or mixed methods study included in the review (N=37).

Multimedia Appendix 6 The Critical Appraisal Skills Program (CASP) qualitative research checklist.

Multimedia Appendix 7 A table of quotes from the included studies illustrating each theme and subtheme identified within the data.

## Abbreviations

CASP: Critical Appraisal Skills Program
CBT: cognitive behavioral therapy
DHI: digital health intervention
ENTREQ: Enhancing Transparency in Reporting the Synthesis of Qualitative Research
HCP: health care professional
QoL: quality of life
PRISMA: Preferred Reporting Items for Systematic Reviews and Meta-Analyses
SPIDER: sample, phenomenon of interest, design, evaluation, and research

## Data Availability

All data generated or analyzed during this study are included in this published article (and its supplementary information files). Additional material can be obtained from the corresponding author.
