# Supplementary material for: Patients’ Experiences of Digital Health Interventions for the Self-Management of Chronic Pain: Systematic Review and Thematic Synthesis
Source: J Med Internet Res. 2025 Mar 18;27:e69100. doi: 10.2196/69100 (PMC11962327; doi:10.2196/69100)
Supplement: Multimedia Appendix 2 [file jmir_v27i1e69100_app2.doc]

Multimedia appendix 2: Search Strategy for Each Database.

**EMBASE (Ovid)**

1. chronic pain/
2. (chronic adj5 pain).mp.
3. (chronic pain or persistent pain or long-term pain or fibromyalgia or rheumatoid arthritis or neuro* pain or musculoskeletal pain* or orofacial pain or visceral pain or endometriosis or headache or irritable bowel syndrome or back pain* or low back pain or neck pain or joint pain* sciatica or osteoarthritis or cancer pain or post surg* pain or post trauma* pain).mp.
4. Telemedicine/
5. telehealth/
6. (health adj5 technolog*).mp.
7. (health adj5 app*).mp.
8. (mhealth or ehealth or telenursing or telerehabilitation or digital health or digital intervention or remote consultation or electronic health or internet health).mp.
9. patient satisfaction/
10. patient attitude/
11. (experience* or perspective* or perception* or accept* or satisf* or view* or attitude*).mp.
12. qualitative research/
13. (qualitative or grounded theory or ethnography or thematic analysis or mixed method* or focus group* or interview*).mp.
14. or/1-3
15. or/4-8
16. or/9-11
17. or/12-13
18. and/14-17
19. limit 18 to (english and last 10 years)

**PubMed:**

((("chronic pain"[Title/Abstract] OR "persistent pain"[Title/Abstract] OR "long-term pain"[Title/Abstract] OR fibromyalgia[Title/Abstract] OR "rheumatoid arthritis"[Title/Abstract] OR osteoarthritis[Title/Abstract] OR "neuro* pain"[Title/Abstract] OR "musculoskeletal pain*"[Title/Abstract] OR "orofacial pain"[Title/Abstract] OR "visceral pain"[Title/Abstract] OR endometriosis[Title/Abstract] OR headache[Title/Abstract] OR "irritable bowel syndrome"[Title/Abstract] OR "back pain*"[Title/Abstract] OR "low* back pain"[Title/Abstract] OR "neck pain"[Title/Abstract] OR "joint pain*"[Title/Abstract] OR sciatica[Title/Abstract] OR "cancer pain"[Title/Abstract] OR "post surg* pain"[Title/Abstract] OR "post trauma* pain"[Title/Abstract] OR "complex regional pain"[Title/Abstract] OR "chronic primary headache"[Title/Abstract] OR "chronic primary visceral pain"[Title/Abstract] OR "chronic musculoskeletal pain"[Title/Abstract])

AND

(experience*[Title/Abstract] OR perspective*[Title/Abstract] OR perception*[Title/Abstract] OR accept*[Title/Abstract] OR satisf*[Title/Abstract] OR view*[Title/Abstract] OR attitude*[Title/Abstract]))

AND

(qualitative[Title/Abstract] OR "grounded theory"[Title/Abstract] OR ethnography[Title/Abstract] OR "thematic analysis"[Title/Abstract] OR "mixed method*"[Title/Abstract] OR "focus group*"[Title/Abstract] OR interview*[Title/Abstract]))

AND

(mhealth[Title/Abstract] OR ehealth[Title/Abstract] OR telenursing[Title/Abstract] OR telerehabilitation[Title/Abstract] OR "digital intervention"[Title/Abstract] OR "remote consultation"[Title/Abstract] OR "electronic health"[Title/Abstract] OR "internet health"[Title/Abstract] OR "digital health"[Title/Abstract] OR "health technolog*"[Title/Abstract] OR "health management app*"[Title/Abstract] OR "digital health program*"[Title/Abstract])

Limit to last 10 years and English language

**Medline (Ovid)**

1. chronic pain/
2. (chronic adj5 pain).mp.
3. (chronic pain or persistent pain or long-term pain or fibromyalgia or rheumatoid arthritis or neuro* pain or musculoskeletal pain* or orofacial pain or visceral pain or endometriosis or headache or irritable bowel syndrome or back pain* or low back pain or neck pain or joint pain* sciatica or osteoarthritis or cancer pain or post surg* pain or post trauma* pain).mp.
4. Telemedicine/
5. (health adj5 technolog*).mp.
6. (health adj5 app*).mp.
7. (mhealth or ehealth or telenursing or telerehabilitation or digital health or digital intervention or remote consultation or electronic health or internet health).mp.
8. Patient Satisfaction/
9. Attitude to Health/
10. (experience* or perspective* or perception* or accept* or satisf* or view* or attitude*).mp.
11. qualitative research/
12. (qualitative or grounded theory or ethnography or thematic analysis or mixed method* or focus group* or interview*).mp.
13. or/1-3
14. or/4-7
15. or/8-10
16. or/11-12
17. and/13-16
18. limit 17 to (english and last 10 years)

**CINAHL (EBSCOhost)**

- MH "chronic pain" OR TI ( "chronic pain" or "persistent pain" or "long-term pain" or fibromyalgia or "rheumatoid arthritis" or osteoarthritis or "neuro* pain" or "musculoskeletal pain*" or "orofacial pain" or "visceral pain" or endometriosis or headache or "irritable bowel syndrome" or "back pain*" or "low* back pain" or "neck pain" or "joint pain*" or sciatica or "cancer pain" or "post surg* pain" or "post trauma* pain" or "complex regional pain" or "chronic primary headache" or "chronic primary visceral pain" or "chronic musculoskeletal pain" ) OR AB ( "chronic pain" or "persistent pain" or "long-term pain" or fibromyalgia or "rheumatoid arthritis" or osteoarthritis or "neuro* pain" or "musculoskeletal pain*" or "orofacial pain" or "visceral pain" or endometriosis or headache or "irritable bowel syndrome" or "back pain*" or "low* back pain" or "neck pain" or "joint pain*" or sciatica or "cancer pain" or "post surg* pain" or "post trauma* pain" or "complex regional pain" or "chronic primary headache" or "chronic primary visceral pain" or "chronic musculoskeletal pain" ) OR SU ( "chronic pain" or "persistent pain" or "long-term pain" or fibromyalgia or "rheumatoid arthritis" or osteoarthritis or "neuro* pain" or "musculoskeletal pain*" or "orofacial pain" or "visceral pain" or endometriosis or headache or "irritable bowel syndrome" or "back pain*" or "low* back pain" or "neck pain" or "joint pain*" or sciatica or "cancer pain" or "post surg* pain" or "post trauma* pain" or "complex regional pain" or "chronic primary headache" or "chronic primary visceral pain" or "chronic musculoskeletal pain" )

AND

MH ( "telehealth" or "digital health" ) OR TI ( mhealth or ehealth or telenursing or telerehabilitation or "digital intervention" or "remote consultation" or "electronic health" or "internet health" or "digital health" or “health technolog*" OR "health management app*" OR "digital health programme" OR "digital health app*" ) OR AB ( mhealth or ehealth or telenursing or telerehabilitation or "digital intervention" or "remote consultation" or "electronic health" or "internet health" or "digital health" or “health technolog*" OR "health management app*" OR "digital health programme" OR "digital health app*" ) OR SU ( mhealth or ehealth or telenursing or telerehabilitation or "digital intervention" or "remote consultation" or "electronic health" or "internet health" or "digital health" or “health technolog*" OR "health management app*" OR "digital health programme" OR "digital health app*" )

AND

MH ("patient satisfaction" or "patient attitudes") OR TI (experience* or perspective* or perception* or accept* or satisf* or view* or attitude* ) OR AB (experience* or perspective* or perception* or accept* or satisf* or view* or attitude*) OR SU (experience* or perspect* or perception* or accept* or satisf* or view* or attitude*)

AND

MH qualitative research OR TI ( qualitative or "grounded theory" or ethnography or "thematic analysis" or "mixed method*" or "focus group*" or interview* ) OR AB ( qualitative or "grounded theory" or ethnography or "thematic analysis" or "mixed method*" or "focus group*" or interview* ) OR SU ( qualitative or "grounded theory" or ethnography or "thematic analysis" or "mixed method*" or "focus group*" or interview* )

 Limit to last 10 years and English language

**PsychInfo (EBSCOhost)**

MH "chronic pain" OR TI ( "chronic pain" or "persistent pain" or "long-term pain" or fibromyalgia or "rheumatoid arthritis" or osteoarthritis or "neuro* pain" or "musculoskeletal pain*" or "orofacial pain" or "visceral pain" or endometriosis or headache or "irritable bowel syndrome" or "back pain*" or "low* back pain" or "neck pain" or "joint pain*" or sciatica or "cancer pain" or "post surg* pain" or "post trauma* pain" or "complex regional pain" or "chronic primary headache" or "chronic primary visceral pain" or "chronic musculoskeletal pain" ) OR AB ( "chronic pain" or "persistent pain" or "long-term pain" or fibromyalgia or "rheumatoid arthritis" or osteoarthritis or "neuro* pain" or "musculoskeletal pain*" or "orofacial pain" or "visceral pain" or endometriosis or headache or "irritable bowel syndrome" or "back pain*" or "low* back pain" or "neck pain" or "joint pain*" or sciatica or "cancer pain" or "post surg* pain" or "post trauma* pain" or "complex regional pain" or "chronic primary headache" or "chronic primary visceral pain" or "chronic musculoskeletal pain" ) OR SU ( "chronic pain" or "persistent pain" or "long-term pain" or fibromyalgia or "rheumatoid arthritis" or osteoarthritis or "neuro* pain" or "musculoskeletal pain*" or "orofacial pain" or "visceral pain" or endometriosis or headache or "irritable bowel syndrome" or "back pain*" or "low* back pain" or "neck pain" or "joint pain*" or sciatica or "cancer pain" or "post surg* pain" or "post trauma* pain" or "complex regional pain" or "chronic primary headache" or "chronic primary visceral pain" or "chronic musculoskeletal pain" )

AND

MH ( "telemedicine" or "mobile health" ) OR TI ( mhealth or ehealth or telenursing or telerehabilitation or "digital intervention" or "remote consultation" or "electronic health" or "internet health" or "digital health" or “health technolog*" OR "health management app*" OR "digital health programme" OR "digital health app*" ) OR AB ( mhealth or ehealth or telenursing or telerehabilitation or "digital intervention" or "remote consultation" or "electronic health" or "internet health" or "digital health" or “health technolog*" OR "health management app*" OR "digital health programme" OR "digital health app*" ) OR SU ( mhealth or ehealth or telenursing or telerehabilitation or "digital intervention" or "remote consultation" or "electronic health" or "internet health" or "digital health" or “health technolog*" OR "health management app*" OR "digital health programme" OR "digital health app*" )

AND

MH ( "patient satisfaction" ) OR TI ( experience* or perspective* or perception* or accept* or satisf* or view* or attitude* ) OR AB ( experience* or perspective* or perception* or accept* or satisf* or view* or attitude* ) OR SU ( experience* or perspective* or perception* or accept* or satisf* or view* or attitude* )

AND

MH qualitative research OR TI ( qualitative or "grounded theory" or ethnography or "thematic analysis" or "mixed method*" or "focus group*" or interview* ) OR AB ( qualitative or "grounded theory" or ethnography or "thematic analysis" or "mixed method*" or "focus group*" or interview* ) OR SU ( qualitative or "grounded theory" or ethnography or "thematic analysis" or "mixed method*" or "focus group*" or interview* )

Limit to last 10 years and English language

**SCOPUS**

( TITLE-ABS-KEY ( "chronic pain" OR "persistent pain" OR "long-term pain" OR fibromyalgia OR "rheumatoid arthritis" OR osteoarthritis OR "neuro* pain" OR "musculoskeletal pain*" OR "orofacial pain" OR "visceral pain" OR endometriosis OR headache OR "irritable bowel syndrome" OR "back pain*" OR "low* back pain" OR "neck pain" OR "joint pain*" OR sciatica OR "cancer pain" OR "post surg* pain" OR "post trauma* pain" OR "complex regional pain" OR "chronic primary headache" OR "chronic primary visceral pain" OR "chronic musculoskeletal pain" )

AND

TITLE-ABS-KEY ( experience* OR perspective* OR perception* OR accept* OR satisf* OR view* OR attitude* )

AND

TITLE-ABS-KEY ( qualitative OR "grounded theory" OR ethnography OR "thematic analysis" OR "mixed method*" OR "focus group*" OR interview* )

AND

TITLE-ABS-KEY ( mhealth OR ehealth OR telenursing OR telerehabilitation OR "digital intervention" OR "remote consultation" OR "electronic health" OR "internet health" OR "digital health" OR "health technolog*" OR "health management app*" OR "digital health program*" ) )

AND

PUBYEAR > 2013 AND PUBYEAR < 2023 AND ( LIMIT-TO ( LANGUAGE , "english" ) )
