# Supplementary material for: Patients’ Experiences of Digital Health Interventions for the Self-Management of Chronic Pain: Systematic Review and Thematic Synthesis
Source: J Med Internet Res. 2025 Mar 18;27:e69100. doi: 10.2196/69100 (PMC11962327; doi:10.2196/69100)
Supplement: Multimedia Appendix 4 [file jmir_v27i1e69100_app4.doc]

Multimedia Appendix 4 – Summary of study characteristics from each qualitative or mixed-method study included in the review (N=37).

| **Author, year, country** | **Study primary aim** | **Study design** | **Key findings/overarching themes** | **Participant demographics** | **Data collection method** |
| --- | --- | --- | --- | --- | --- |
| Algeo et al, 2017, United Kingdom [44] | To explore the attitudes of a PPI group towards using ehealth for joint pain management. | Qualitative | - Attitudes: - Positive attitudes towards using e-health to self-manage joint pain - Early disease support - Facilitators: - Personalised action plan - Prompting - Interactive aspects - Trustworthiness - Barriers: - Excess information | 10 PPI members  7F, 3M  42-74  Hip and/or knee osteoarthritis | Telephone interviews  Thematic analysis |
| Arensman et al, 2022, Netherlands [45] | To explore acceptability, satisfaction and performance of a pain management app. | Qualitative | - Acceptability - Satisfaction - Performance | 9 participants  5F, 4M  20-70  Non-specific low back pain | In person interviews  Framework method |
| Austin et al, 2020, United Kingdom [46] | To explore acceptability and feasibility of collecting patient-generated health data using smartphones. | Qualitative | - RA as an ‘invisible disease’ - Providing the ‘bigger picture’ of RA - Enabling person-centred consultations | 17 participants  Rheumatoid Arthritis | Interviews  Thematic analysis |
| Ball et al, 2020, United Kingdom [47] | To determine acceptability, usability, and feasibility of a mindfulness meditation smartphone application for chronic pelvic pain. | Qualitative | - Familiarity and capabilities with app technology. - Motivations to use the app. - Perceived benefits. - Relation to other therapies. - Opportunities to use the app. - Technology issues getting in the way. - Life getting in the way. | 14 participants  14F  Chronic pelvic pain | Telephone and in-person interviews  Thematic analysis |
| Bostrom et al, 2022, Norway [48] | To explore the experiences of engaging with EPIO for individuals with chronic pain. | Qualitative | - Engaging with EPIO - Coping with pain in everyday life - The value of engaging with the EPIO program | 15 participants  8F, 7M  43-74  Chronic pain in general | Telephone interviews  Thematic analysis |
| Cronstrom et al, 2019, Sweden [49] | To investigate the patients’ experiences of using a digital management programme for hip and knee OA. | Qualitative | - Management options for mitigating the consequences of OA - Experiences of the digital programme - Perceived effects of the digital programme over time. | 19 participants  10F, 9M  45-80  Knee or hip osteoarthritis | Face-to-face, telephone and Skype interviews  Systematic text condensation |
| De Groef et al, 2023, Belgium [75] | To evaluate the eHealth intervention’s acceptability, comprehensibility, and satisfaction. | Mixed methods | - Usability - Visual design - User engagement - Content - Therapeutic persuasiveness - Therapeutic alliance - Overall evaluation | 29 participants  29F  Mean age 50.8, SD (11.6)  Breast cancer  survivors with persistent pain | Online focus groups  Framework method |
| Garrett, Taverner & McDade, 2017, Canada [50] | To explore patient’s perceptions of their pain while using VR, if they observed any practical application of safety issues in using VR or experienced any adverse effects. | Mixed methods | - Design of the VR experiences - Efficacy of VR for chronic pain - Limits of the VR technology - Practicality of use as an adjunctive therapy | 8 participants  6F, 2M  31-71  Chronic pain in general | In person interview  Interpretive-description approach |
| Garrett et al, 2020, Canada [77] | To explore cancer patients with chronic pain experiences and perceptions of using home-based VR for pain management. | Qualitative | - Activities, - Usability - Effects - Mode of action - Technical aspects | 12 participants  4F, 6M, 2 undisclosed  37-73  Cancer patients with chronic pain | In-person and online focus groups  Interpretive-description approach |
| Godziuk et al, 2023, Canada [51] | To evaluate acceptability and effectiveness of a self-care intervention for knee osteoarthritis. | Mixed methods | - Acceptability - Tailored and reliable information - Preferences for online or offline content. - Engagement - Preliminary effectiveness | 15 participants  Knee osteoarthritis | Telephone or videocall interviews  Content analysis |
| Grolier et al, 2023, France [78] | To refine the design of a self-management smartphone application for chronic non-specific lower back pain. | Mixed methods | Participants agreed that the intervention was useful and motivating. Participants wanted to use the app and stated they would recommend to a friend. Suggested adding music and videos, as well as additional exercises. | 18 participants  9F, 9M  23-53  Chronic non-specific lower back pain | Questionnaires  Content analysis |
| Hogan et al, 2022, USA [52] | To evaluate user experiences with the Pain Coach app and its impact on pain-related outcomes. | Mixed methods | - Veteran-Reported Use of the Pain Coach App - Patterns of Use and Usability of the Pain Coach App - Usefulness, Communication, and Outcomes Associated with Pain Coach App Use - Reasons for Non-use | 10 veterans  Chronic pain in general | Telephone interviews  Thematic analysis |
| Hoving et al, 2014, Netherlands [79] | To determine whether an e-health programme could work in a rheumatology setting, as an adjunct to standard care. | Qualitative | Participants were satisfied with the programme. Participants found the website user friendly. Suggested improvements were to add more questions, change the layout and have a section for sharing experiences. | 15 participants  Rheumatoid arthritis | Questionnaires and interviews  Thematic analysis |
| Jeon et al, 2019, Australia [53] | To explore the experiences and perceptions of users of the OA-Hub concerning OA self-management and decision support. | Qualitative | - Gaining new insights and empowerment - Accessing quality, sensible and balanced information through an authoritative voice. - Preserving and Tracking information | 36 participants  40-86  Knee and/or hip osteoarthritis | Telephone interviews  Content analysis |
| Kawi et al, 2022, USA [54] | To explore the feasibility of vAPA in the self-management of chronic pain. | Qualitative | - vAPA was feasible. Other themes were: - Better control of pain - Less use of pain medications - Self-management and motivation in pain - Expectations for pain relief | 18 participants  13F, 5M  24-80  Chronic pain in general | In person interviews  Content analysis |
| Knoerl et al, 2022, USA [55] | To explore cancer survivors' perspectives of the acceptability and a virtual yoga program for chronic chemotherapy induced peripheral neuropathy pain. | Mixed methods | - Cancer Survivors With Chronic Chemotherapy-Induced Peripheral Neuropathy Pain Desire New Treatments - The Impact of Yoga on Chemotherapy-Induced Peripheral Neuropathy Is Unclear - Acceptability and Satisfaction With Virtual Yoga Practice | 14 participants  14F  39-74  Cancer Survivors With Chronic Chemotherapy- Induced Peripheral Neuropathy Pain | Telephone interviews  Content analysis |
| Lamper et al, 2021, Netherlands [56] | To explore the feasibility of the eCoach-Pain for patients and PHCPs. | Mixed methods | - Overall Opinion and Usage - Pain Complexity Tool and Diaries - Educational Sessions - Chat Function and Communication with PHCP - Technical Issues - Future Usage and Recommendations of the eCoach-Pain | 29 patients  21F, 8M  24-71  Chronic musculoskeletal pain | Telephone interviews  Thematic analysis |
| Merolli, Gray & Martin-Sanchez, 2016, Australia [57] | To examine therapeutic affordances of social media that were described by patients within a clinical program of chronic pain management | Qualitative | - Therapeutic affordances - Barriers to participation | 17 participants  10F, 7M  18-50  Chronic pain in general | Telephone interviews  Thematic content analysis |
| Muehlensiepen et al, 2023, Germany [58] | To investigate the benefit of using an ePRO web-app to support shared decision-making (SDM) and treat-to-target (T2T) in RA patients. | Qualitative | - App user experiences - Perceived drawbacks of app-supported rheumatology care - Perceived benefits of app-supported rheumatology care. | 10 participants  5F, 5M  27-73  Rheumatoid arthritis | Telephone interviews  Content analysis |
| Nelligan et al, 2020, Australia [59] | To explore the attitudes and experiences of people with knee OA who accessed the self-directed eHealth intervention and the features perceived as useful to facilitate self-directed exercise. | Qualitative | - Technology Easy to Use and Follow - Facilitators to Exercise Participation - Sense of Support and Accountability - Positive outcomes - Suggestions for Real-World Application | 16 participants  8F, 8M  48-75  Knee osteoarthritis | Telephone interviews  Thematic analysis |
| Nordin et al, 2017, Sweden [60] | To explore patients’ experiences of participation in the Web-BCPA intervention in combination with multimodal pain rehabilitation in primary health care. | Qualitative | - One theme: “It’s about me”   Containing 4 categories:   - Take part in a flexible framework of own priority - Acquire knowledge and insights - Ways toward change - Personal and environmental conditions influencing participation | 19 participants  15F, 4M  Mean age 45  Musculoskeletal pain | In person Interviews  Content analysis |
| Östlind et al, 2022, Sweden [73] | To explore the experiences of using a wearable activity tracker to monitor physical activity and the general perceptions of digital support in OA care among individuals of working age with hip and knee osteoarthritis. | Qualitative | - A WAT may aid in optimization of PA but is not a panacea - WATs facilitate PA - Increased awareness of one’s limitations - WATs are not always encouraging - Digital support is an appreciated part of OA care - Individualized, early and continuous support - PT is essential but needs to be modernized - Easy, comprehensive, and reliable digital support | 18 participants  13F, 5M  Mean age 58, SD (6)  Hip and knee osteoarthritis | In person focus groups  Content analysis |
| Overton et al, 2023, New Zealand [74] | To explore participant experiences and perceptions of using smartphone EMA as a way of communicating knee OA pain and symptoms following participating in a 2-week smartphone EMA study. | Qualitative | - User experience of smartphone EMA - Data quality of smartphone EMA - Practical aspects of smartphone EMA. | 20 participants  12F, 8M  Mean age 71.1, SD (4.2)  Knee osteoarthritis | In person focus groups  Thematic analysis |
| Rini et al, 2018, USA [61] | To collect feedback from patients who completed the program and to understand their views of current cancer pain treatments, PCST (including web based PCST), and ways to support its use in clinical care. | Qualitative | - Pain in Everyday Life - Experiences with Web-Based PCST - Changes resulting from use of web-based PCST | 7 participants  4F, 3M  56-70  Persistent bone pain due to multiple myeloma or metastasized breast or prostate cancer | In person interviews  Content analysis |
| Schlett et al, 2022, Germany [62] | To investigate the acceptance, usability, and utility of the web portal tala-med from the patient. | Qualitative | - Primary care physicians use of the portal during the consultation - Patient use of the portal - Usability - Added value - Effects of the portal | 32 participants  16F, 16M  18-79  Chronic low back pain | Telephone interviews  Framework analysis |
| Seppen et al, 2020, Netherlands [80] | To explore the feasibility of the MijnReuma Reade smartphone application for the self-monitoring of rheumatoid arthritis. | Mixed methods | - Facilitators:   Gaining control over their disease  Improved communication with HCP   - Barriers:   Less pain | 27 participants  21F, 6M  Mean age 52, SD (11)  Rheumatoid arthritis | Questionnaires and telephone interviews  Thematic analysis |
| Seppen et al, 2023, Netherlands [76] | To investigate what the barriers and facilitators are to adhere to weekly monitoring of disease activity with ePROs in patients with inflammatory arthritis with the aim to identify factors that could be used to improve adherence. | Qualitative | - Questionnaire frequency - Discussing results of completed ePROs - Physical consultations - Patient insight into disease activity - User experience of the app | 22 participants  12F, 10M  Mean age 64, SD (10)  rheumatoid arthritis (RA), psoriatic arthritis (PsA), or ankylosing spondylitis (AS) | Online focus groups  Thematic analysis |
| Shewchuk et al, 2021, Canada [63] | To evaluate the overall usability, quality, and effectiveness of the mHealth app prototype for aiding knee OA self-management from the perspectives of patients with OA and health care providers (HCPs). | Mixed methods | - Usefulness - Ease of use - Contribution to OA knowledge - Self-management potential - Perceptions of the impact on patient-HCP communication | 18 participants  11F, 7M  Mean age 66.2, SD (6.9)  Early knee osteoarthritis | Telephone and in-person interviews  Thematic analysis and descriptive analysis |
| Stern et al, 2022, USA [64] | To explore patients’ perspectives on the benefits of receiving feedback on PROMs in the context of a web-based personalised decision report to guide care for their hip or knee osteoarthritis. | Qualitative | - Gaining Information About My Health Status - Fostering Communication Between Patient and Surgeon - Increasing My Confidence and Trust | 25 participants  15F, 10M  49-82  Hip and/or knee osteoarthritis | Telephone or video interviews  Qualitative descriptive approach |
| Svanholm et al, 2023, Sweden [65] | To describe the acceptability of SWEPPE after IPRPs from the perspective of patients with chronic pain and their employers. | Mixed methods | - Affective attitude - Perceived effectiveness - Intervention coherence - Self-efficacy - Burden - Ethicality | 11 participants  10F, 1M  Mean age 42.5, SD (5.2)  Chronic pain | Telephone or video interviews  Content analysis |
| Svendsen et al, 2022, Denmark and Norway [66] | To investigate the experiences of patients who participated in the selfBACK randomised controlled trial. | Qualitative | - Facilitators: preferences and beliefs regarding self-management, a supporter within the intervention that is friendly and motivating. Content that is tailored and personalised, is convenient, easy to use, and ease of use, and the user can see the benefit. - Key impeding factors: not having belief in self-management, technical issues, not having enough time, obtaining no benefit from the intervention, not enough contact with a HCP. | 26 participants  11F, 15M  21-78  Chronic lower back pain | Telephone interviews  Framework analysis |
| Tonga et al, 2021, Turkey [67] | To develop a mHealth app using an iterative user-centred design approach to ensure the product met user needs, was easy to use and acceptable to both patients with RA and therapists and test its usability. | Mixed methods | - Learning and accuracy - Ease of use - Motivation and adherence | 17 participants  16F, 1M  39-61  Rheumatoid arthritis | In-person interviews  Thematic analysis |
| Tonkin-Crine et al, 2013, United Kingdom [68] | To explore patients’ views and experiences of using the CBT-based website to facilitate self-management of IBS. | Qualitative | - IBS is unpredictable and uncontrollable - Perceptions of medication as a treatment for IBS - Website format is acceptable - Engagement with the Regul8 website. | 31 participants  16F, 15M  Mean age 51, SD (8.6)  Irritable bowel syndrome | Telephone interviews  Thematic analysis |
| Van Der Meer et al, 2022, Netherlands [69] | To assess the experience and perceived additional value of e-Health, using the Physitrack application, during the physical therapy treatment for patients with TMD. | Mixed methods | - The intervention is perceived as helpful, can provide personalised care, contains satisfying content and is friendly for users, is accessible, efficient and motivating. - Participants advised that shared decision-making was an important feature. - Found that exercise videos added value to traditional care. | 10 participants  10F  Mean age 52.3  Temporomandibular disorder | In-person and online interviews |
| Whitney et al, 2018, USA [70] | To explore patients’ experiences in monitoring their own symptoms and collaborating with primary care providers to use PGHD resulting from mHealth supported N-of-1 trials for chronic pain management. | Qualitative | - Patients were enthusiastic about *accessing* their PGHD. - Patients found value in *sharing* data with their clinicians. - Patients engaged energetically in *using* their own data for a wide range of purposes, some apart from those anticipated by investigators. | 33 participants  15F, 18M  Mean age 55, SD (10)  Chronic musculoskeletal pain | Interviews  Thematic analysis |
| Yeh et al, 2022, USA [71] | To test the feasibility of technology using a smartphone app as a self-guided tool to help people learn and self-administer APA to manage cLBP. Specifically, this study assessed (1) the feasibility (i.e., recruitment, adherence to APA, acceptability), and (2) cLBP outcomes between two groups (app vs app + telehealth). | Mixed methods | - The APA app was found to be: - Acceptable - Feasible - Supportive | 31 participants  Chronic low back pain | In-person or video interviews  Content analysis |
| Zuidema et al, 2019, Netherlands [72] | To explore use and non-use of an online pain management programme as well as the experience of the online programme amongst users. | Qualitative | - Not all patients were motivated to use the program - patients had no clear expectation or had differing expectations of the program - there was a mismatch between individual patients’ support needs and the needs included in the program - reminders were only sent to fill in the diaries for pain and fatigue, not to use the program modules. | 21 participants  13F, 8M  44-78  Rheumatoid arthritis | Telephone interviews  Thematic analysis |

APA – auricular point acupressure; CBT – cognitive behavioural therapy; cLBP – chronic lower back pain; e-Health – electronic-health; EMA – ecological momentary assessment; HCP – healthcare professional; IBS – irritable bowel syndrome; IPRPs – Interdisciplinary pain rehabilitation programs; OA – osteoarthritis; PCST – pain coping skills training; PPI – patient and public involvement; RA – rheumatoid arthritis; TMD - temporomandibular disorder; vAPA – virtual auricular point acupressure; VR – virtual reality.

**Reference list:**

44. Algeo N, Hunter D, Cahill A, Dickson C, Adams J. Usability of a digital self-management website for people with osteoarthritis: A UK patient and public involvement study. International Journal of Therapy & Rehabilitation. 2017;24(2):78-82. PMID: 121447740. Language: English. Entry Date: 20170228. Revision Date: 20170303. Publication Type: Article. doi: 10.12968/ijtr.2017.24.2.78.

45. Arensman R, Kloek C, Pisters M, Koppenaal T, Ostelo R, Veenhof C. Patient Perspectives on Using a Smartphone App to Support Home-Based Exercise During Physical Therapy Treatment: Qualitative Study. JMIR Human Factors. 2022 2022;9(3). PMID: rayyan-562659184. doi: doi:10.2196/35316.

46. Austin L, Sharp CA, van der Veer SN, Machin M, Humphreys J, Mellor P, et al. Providing 'the bigger picture': Benefits and feasibility of integrating remote monitoring from smartphones into the electronic health record. Rheumatology (United Kingdom). 2020;59(2):367-78. PMID: 632830193.

47. Ball E, Newton S, Rohricht F, Steed L, Birch J, Dodds J, et al. MHealth: providing a mindfulness app for women with chronic pelvic pain in gynaecology outpatient clinics: qualitative data analysis of user experience and lessons learnt. BMJ Open. 2020 2020-3-12;10. PMID: rayyan-562659189.

48. Bostrom K, Varsi C, Eide H, Borosund E, Kristjansdottir OB, Schreurs KMG, et al. Engaging with EPIO, a digital pain self-management program: a qualitative study. BMC health services research. 2022 2022-4-29;22:577. PMID: rayyan-562659211.

49. Cronström A, Dahlberg LE, Nero H, Ericson J, Hammarlund CS. 'I would never have done it if it hadn't been digital': a qualitative study on patients' experiences of a digital management programme for hip and knee osteoarthritis in Sweden. BMJ Open. 2019 May 24;9(5):e028388. PMID: 31129601. doi: 10.1136/bmjopen-2018-028388.

50. Garrett B, Taverner T, McDade P. Virtual Reality as an Adjunct Home Therapy in Chronic Pain Management: An Exploratory Study. JMIR Medical Informatics. 2017;5(2):e11. PMID: 28495661.

51. Godziuk K, Prado CM, Quintanilha M, Forhan M. Acceptability and preliminary effectiveness of a single-arm 12-week digital behavioral health intervention in patients with knee osteoarthritis. BMC Musculoskeletal Disorders. 2023 2023-12;24. PMID: rayyan-562659279.

52. Hogan TP, Etingen B, McMahon N, Bixler FR, Am L, Wacks RE, et al. Understanding Adoption and Preliminary Effectiveness of a Mobile App for Chronic Pain Management Among US Military Veterans: Pre-Post Mixed Methods Evaluation. JMIR Formative Research. 2022 2022;6(1):e33716. PMID: rayyan-540241597.

53. Jeon YH, Flaherty I, Urban H, Wortley S, Dickson C, Salkeld G, et al. Qualitative Evaluation of Evidence-Based Online Decision Aid and Resources for Osteoarthritis Management: Understanding Patient Perspectives. Arthritis Care and Research. 2019 2019-1;71:46-55. PMID: rayyan-562659323.

54. Kawi J, Yeh CH, Lukkahatai N, Hardwicke RL, Murphy T, Christo PJ. Exploring the Feasibility of Virtually Delivered Auricular Point Acupressure in Self-Managing Chronic Pain: Qualitative Study. Evidence-based Complementary and Alternative Medicine. 2022 2022;2022. PMID: rayyan-562659330.

55. Knoerl R, Bockhoff J, Fox E, Giobbie-Hurder A, Berry DL, Berfield J, et al. Cancer Survivors' Perspectives of Virtual Yoga for Chronic Chemotherapy-Induced Peripheral Neuropathy Pain During the COVID-19 Pandemic. CIN: Computers, Informatics, Nursing. 2022;40(9):641-7. PMID: 159061718. Language: English. Entry Date: 20221003. Revision Date: 20230308. Publication Type: Article. doi: 10.1097/CIN.0000000000000937.

56. Lamper C, Huijnen I, de Mooij M, Koke A, Verbunt J, Kroese M. An eCoach-Pain for Patients with Chronic Musculoskeletal Pain in Interdisciplinary Primary Care: A Feasibility Study. International Journal of Environmental Research & Public Health [Electronic Resource]. 2021;18(21):06. PMID: 34770177.

57. Merolli M, Gray K, Martin-Sanchez F. Patient Participation in Chronic Pain Management Through Social Media: A Clinical Study. Studies in health technology and informatics. 2016 2016;225:577-81. PMID: rayyan-562659379.

58. Muehlensiepen F, May S, Hadaschik K, Vuillerme N, Heinze M, Grahammer M, et al. Digitally supported shared decision-making and treat-to-target in rheumatology: a qualitative study embedded in a multicenter randomized controlled trial. Rheumatology International. 2023 2023-4;43:695-703. PMID: rayyan-562659390.

59. Nelligan RK, Hinman RS, Teo PL, Bennell KL. Exploring attitudes and experiences of people with knee osteoarthritis toward a self-directed ehealth intervention to support exercise: Qualitative Study. JMIR Rehabilitation and Assistive Technologies. 2020 2020;7(2). PMID: rayyan-562659401. doi: doi:10.2196/18860.

60. Nordin C, Michaelson P, Eriksson MK, Gard G. It's About Me: Patients' Experiences of Patient Participation in the Web Behavior Change Program for Activity in Combination With Multimodal Pain Rehabilitation. Journal of medical Internet research. 2017 2017-1-18;19:e22. PMID: rayyan-562659407.

61. Rini C, Vu MB, Lerner H, Bloom C, Carda-Auten J, Wood WA, et al. A qualitative study of patient and provider perspectives on using web-based pain coping skills training to treat persistent cancer pain. Palliative & supportive care. 2018 2018-4-1;16:155-69. PMID: rayyan-562659444.

62. Schlett C, Röttele N, van der Keylen P, Schöpf-Lazzarino AC, Klimmek M, Körner M, et al. The Acceptance, Usability, and Utility of a Web Portal for Back Pain as Recommended by Primary Care Physicians: Qualitative Interview Study with Patients. JMIR Formative Research. 2022 2022;6(12). PMID: rayyan-562659458. doi: doi:10.2196/38748.

63. Shewchuk B, Green LA, Barber T, Miller J, Teare S, Campbell-Scherer D, et al. Patients' use of mobile health for self-management of knee osteoarthritis: Results of a 6-week pilot study. JMIR Formative Research. 2021 2021;5(11). PMID: rayyan-562659468. doi: doi:10.2196/30495.

64. Stern BZ, Pila S, Joseph LI, Rothrock NE, Franklin PD. Patients' perspectives on the benefits of feedback on patient-reported outcome measures in a web-based personalized decision report for hip and knee osteoarthritis. BMC Musculoskeletal Disorders. 2022 2022-12;23. PMID: rayyan-562659485.

65. Svanholm F, Turesson C, Löfgren M, Björk M. Acceptability of the eHealth Intervention Sustainable Worker Digital Support for Persons With Chronic Pain and Their Employers (SWEPPE): Questionnaire and Interview Study. JMIR Hum Factors. 2023 Sep 28;10:e46878. PMID: 37768708. doi: 10.2196/46878.

66. Svendsen MJ, Nicholl BI, Mair FS, Wood K, Rasmussen CDN, Stochkendahl MJ. One size does not fit all: Participants' experiences of the selfBACK app to support self-management of low back pain-a qualitative interview study. Chiropractic and Manual Therapies. 2022 2022-12;30. PMID: rayyan-562659488.

67. Tonga E, Williamson E, Srikesavan C, Özen T, Sarıtaş F, Lamb SE. A hand exercise mobile app for people with rheumatoid arthritis in Turkey: design, development and usability study. Rheumatology International. 2021;41(6):1151-60. doi: 10.1007/s00296-021-04860-0.

68. Tonkin-Crine S, Bishop FL, Ellis M, Moss-Morris R, Everitt H. Exploring patients' views of a cognitive behavioral therapy-based website for the self-management of irritable bowel syndrome symptoms. Journal of medical Internet research. 2013 2013;15:e190. PMID: rayyan-562659499. doi: doi:.

69. van der Meer HA, de Pijper L, van Bruxvoort T, Visscher CM, Nijhuis-van der Sanden MWG, Engelbert RHH, et al. Using e-Health in the physical therapeutic care process for patients with temporomandibular disorders: a qualitative study on the perspective of physical therapists and patients. Disability and rehabilitation. 2022 01 Feb;44(4):617-24. PMID: 632104005.

70. Whitney RL, Ward DH, Marois MT, Schmid CH, Sim I, Kravitz RL. Patient perceptions of their own data in mhealth technology–enabled N-of-1 trials for chronic pain: Qualitative study. JMIR mHealth and uHealth. 2018;6(10). doi: 10.2196/10291.

71. Yeh CH, Kawi J, Ni A, Christo P. Evaluating Auricular Point Acupressure for Chronic Low Back Pain Self-Management Using Technology: A Feasibility Study. Pain Management Nursing. 2022;23(3):301-10. PMID: 157501161. Language: English. Entry Date: 20220701. Revision Date: 20220701. Publication Type: Article. doi: 10.1016/j.pmn.2021.11.007.

72. Zuidema RM, Van Dulmen S, Nijhuis-van der Sanden MWG, Fransen J, Van Gaal BGI. qLessons learned from patients with access to an online self-management enhancing program for RA patients: Qualitative analysis of interviews alongside a randomized clinical trial. Patient Educ Couns. 2019 Jun;102(6):1170-7. PMID: 30638903. doi: 10.1016/j.pec.2019.01.005.

73. Östlind E, Ekvall Hansson E, Eek F, Stigmar K. Experiences of activity monitoring and perceptions of digital support among working individuals with hip and knee osteoarthritis – a focus group study. BMC Public Health. 2022;22(1). doi: 10.1186/s12889-022-14065-0.

74. Overton M, Swain N, Falling C, Gwynne-Jones D, Fillingim R, Mani R. Experiences and Perceptions of Using Smartphone Ecological Momentary Assessment for Reporting Knee Osteoarthritis Pain and Symptoms. Clinical Journal of Pain. 2023 19 Sep;39(9):442-51. PMID: 2026479267.

75. De Groef A, Evenepoel M, Van Dijck S, Dams L, Haenen V, Wiles L, et al. Feasibility and pilot testing of a personalized eHealth intervention for pain science education and self-management for breast cancer survivors with persistent pain: a mixed-method study. Supportive Care in Cancer. 2023 2023-2;31. PMID: rayyan-562659237. doi: doi:.

76. Seppen BF, Wiegel J, Nurmohamed MT, Bos WH, ter Wee MM. Facilitators and barriers to adhere to monitoring disease activity with ePROs: a focus group study in patients with inflammatory arthritis. Rheumatology International. 2023 2023-4;43:677-85. PMID: rayyan-562659462. doi: doi:.

77. Garrett BM, Tao G, Taverner T, Cordingley E, Sun C. Patients perceptions of virtual reality therapy in the management of chronic cancer pain. Heliyon. 2020 2020;6(5). PMID: rayyan-562659274. doi: doi:10.1016/j.heliyon.2020.e03916.

78. Grolier M, Arefyev A, Pereira B, Tavares Figueiredo I, Gerbaud L, Coudeyre E. Refining the design of a smartphone application for people with chronic low back pain using mixed quantitative and qualitative approaches. Disability and rehabilitation. 2023 2023-2-1:145-50. PMID: rayyan-562659290.

79. Hoving JL, Zoer I, Van Der Meer M, Van Der Straaten Y, Logtenberg-Rutten C, Kraak-Put S, et al. E-health to improve work functioning in employees with rheumatoid arthritis in rheumatology practice: A feasibility study. Scandinavian Journal of Rheumatology. 2014 01 Nov;43(6):481-7. PMID: 600693450.

80. Seppen BF, Wiegel J, L'Ami M J, Duarte Dos Santos Rico S, Catarinella FS, Turkstra F, et al. Feasibility of Self-Monitoring Rheumatoid Arthritis With a Smartphone App: Results of Two Mixed-Methods Pilot Studies. JMIR Formative Research. 2020;4(9):e20165. PMID: 32955447.
