# Supplementary material for: Patients’ Experiences of Digital Health Interventions for the Self-Management of Chronic Pain: Systematic Review and Thematic Synthesis
Source: J Med Internet Res. 2025 Mar 18;27:e69100. doi: 10.2196/69100 (PMC11962327; doi:10.2196/69100)
Supplement: Multimedia Appendix 5 [file jmir_v27i1e69100_app5.doc]

Multimedia Appendix 5 – Summary of intervention characteristics from each qualitative or mixed-method study included in the review (N=37).

| **Author, year, country** | **Intervention aims** | **Intervention modality** | **Name of digital tool** | **Features of digital tool** | **Frequency and duration of intervention** |
| --- | --- | --- | --- | --- | --- |
| Algeo et al, 2017, United Kingdom [44] | To support and engage patients, and help them develop self-management skills | Website | Myjointpain.org | Goal setting  Action planning  Educational resources  Information resources  Weight management  Physical activity strategies  Progress tracking  HCP locater | 2 weeks |
| Arensman et al, 2022, Netherlands [45] | To support home-based exercise as part of treatment  During treatment | Smartphone application | Physitrack app | Physical activity strategies  Personalised exercised programme  Progress tracking  Pain tracking  HCP communication |  |
| Austin et al, 2020, United Kingdom [46] | To enable patients to monitor their symptoms and impact of disease | Smartphone application | The REMORA system | Symptom tracking and monitoring  Integration into EHR | Daily check-ins |
| Ball et al, 2020, United Kingdom [47] | To facilitate meditation strategies for chronic pelvic pain symptom management | Smartphone application | Modified version of the Headspace meditation app | Mindfulness and meditation strategies  Relaxation techniques | 60 days  10 min for the first 10 days, 15 min up to day 20, and 20 min up to day 60 |
| Bostrom et al, 2022, Norway [48] | To support self-management and well-being for people living with chronic pain in general | Smartphone application | EPIO | CBT techniques  Educational resources  Coping strategies (mindfulness strategies, relaxation techniques, breathing exercises)  Pain tracking  Symptom tracking  Communication and social support | 3-months  Daily tracking, modules open up 3 days after previous module completion |
| Cronstrom et al, 2019, Sweden [49] | To deliver educational content and self-management techniques for people with osteoarthritis | Online programme | Joint Academy | Physical activity,  Weight management,  Educational content  Communication and support | 6-weeks |
| De Groef et al, 2023, Belgium [75] | To provide an understanding of the target concepts, as well as familiarity with cognitive and self-management skills to manage the pain experience and pain-related functioning | Online programme |  | Educational resources, symptom monitoring | 6 weeks |
| Garrett, Taverner & McDade, 2017, Canada [50] | To be used as an adjunctive therapy for chronic pain patients in their own homes | Virtual reality programme with headset |  | Mindfulness and meditation strategies  Problem solving tasks  Gamification elements | 1 month  12 therapeutic sessions of 30 minutes |
| Garrett et al, 2020, Canada [77] | To be used as an adjunctive therapy for chronic pain patients to manage pain in their own homes | Virtual reality |  | Mindfulness and meditation strategies  Problem solving tasks  Gamification elements | 1 month  Six 30-45 minute sessions |
| Godziuk et al, 2023, Canada [51] | To improve access to services and osteo arthritis outcomes | Online platform | My Viva Plan | Nutrition guides  Physical activity strategies  Mindfulness strategies  Goal setting  Tracking and monitoring positive health behaviours | 12-weeks  Programme issued weekly |
| Grolier et al, 2023, France [78] | To support physical exercise training in chronic non-specific lower back pain patients | Smartphone application | eLombactif | Physical activity strategies  Educational resources  Support forum | 3-weeks |
| Hogan et al, 2022, USA [52] | To manage chronic pain in veterans | Smartphone application | VA Pain Coach App | Pain tracking  Educational resources  Motivational messages  Share information with VA care team  Breathing exercises  Relaxation techniques  Activity pacing | 6 months  Daily check-ins |
| Hoving et al, 2014, Netherlands [79] | To help RA patients improve their work functioning | e-health program  Website |  | Problem solving activities  Action plans  Goal setting  Online support | 3-months |
| Jeon et al, 2019, Australia [53] | To help people with OA achieve measurable improvements in health outcomes and health care utilisation | Website | The OA-Hub (contains Myjointpain.org.au) | Treatment comparison  Goal setting  Educational resources  Weight management  Physical activity strategies  Progress tracking  HCP locater | Up to 2-months |
| Kawi et al, 2022, USA [54] | To facilitate at home, self-administered auricular point acupressure | Smartphone application |  | Auricular point acupressure | 4 weeks  3 times a day for 3-minutes |
| Knoerl et al, 2022, USA [55] | To deliver virtual yoga classes using prerecorded videos to relieve chronic CIPN pain | Online programme |  | Yoga exercises  Mindfulness exercises  Breathing exercises | 8-weeks  45 minutes per week |
| Lamper et al, 2021, Netherlands [56] | To support the provision of integrated rehabilitation care for chronic musculoskeletal pain patients  Approximately | Online programme | The eCoach-Pain | Journaling/diary  Pain tracking  Educational resources  Chat function | 3 months  Daily check-ins |
| Merolli, Gray & Martin-Sanchez, 2016, Australia [57] | To use social media resources (including Facebook, YouTube and various chronic pain blogs) to assist chronic pain management | Social media |  | Community forums, blogs  Pain management videos | 12-weeks  As and when required |
| Muehlensiepen et al, 2023, Germany [58] | To collect ePROs to facilitate remote monitoring and self-assessment disease monitoring | Smartphone application | ABATON RA | Pain tracking  Symptom tracking | At least 3-months  Every 3 days |
| Nelligan et al, 2020, Australia [59] | To increase the uptake of healthy behaviours through self-directed exercise videos | Website and SMS programme | My Knee Exercise | Physical activity strategies  Educational/informational resources  Exercise tracking  SMS prompting | 24-weeks  3x per week |
| Nordin et al, 2017, Sweden [60] | Developed to propose an eHealth solution for a biopsychosocial treatment of persistent musculoskeletal pain. | Website | Web-BCPA | CBT behaviour change techniques  Information resources  Educational content and assignments  Coping mechanisms  Physical activity  Nutritional information | 4-months  Access to the website 24/7 |
| Östlind et al, 2022, Sweden [73] | To self-monitor and promote physical activity in hip and knee osteoarthritis | Wearable activity tracker (WAT) | FitBit | Physical activity Exercises  Goal setting | 12-weeks  Daily |
| Overton et al, 2023, New Zealand [74] | To monitor, assess and communicate knee osteoarthritis symptoms | Smartphone application | Smartphone ecological momentary assessment (EMA) | Pain tracking  Physical activity tracking | 2-weeks  3 times daily |
| Rini et al, 2018, USA [61] | To expand access to PCST therapy | Web-based programme | PainCOACH | Muscle relaxation techniques  Physical activity strategies  Coping mechanisms  Distraction techniques  Problem solving  Goal setting | 8-10 weeks  Eight interactive 35- to 45-minute sessions |
| Schlett et al, 2022, Germany [62] | To improve share-decision making and provide high-quality information to patients with chronic low back pain | Web portal | Tala-med | Educational content  Information resources | 1-2 months |
| Seppen et al, 2020, Netherlands [80] | To facilitate patient reported outcome monitoring for rheumatoid arthritis | Smartphone application | The MijnReuma Reade App | Self-assessment questionnaires on pain, sleep, anxiety, and stress | 4-weeks  Weekly data collection |
| Seppen et al, 2023, Netherlands [76] | To investigate the incentives for patients to continue or stop monitoring their disease activity with ePROs over time | Smartphone application | The MyReumatism app | Self-assessment health questionnaires |  |
| Shewchuk et al, 2021, Canada [63] | To support self-management of knee osteoarthritis symptoms | Smartphone application |  | Symptom tracking  Goal setting  Physical activity exercises  Journaling  Activities?  Information resources | 12-weeks  Daily use |
| Stern et al, 2022, USA [64] | To support shared decision making | Web-based |  | Self-assessment health questionnaires  Alternative treatment options | 12-months |
| Svanholm et al, 2023, Sweden [65] | During the IPRPs, patients worked with individual goals and strategies to improve their health and participation in activities and work. | Smartphone application and web application | SWEPPE: n Sustainable Worker Digital Support for Persons with Chronic Pain and Their Employers (SWEPPE) | Action planning  Goal setting  Pain tracking  Activity tracking  Online coach  Information resources  Educational content | 6 and 10 weeks  Daily use |
| Svendsen et al, 2022, Denmark and Norway [66] | selfBACK is an artificial intelligence-based smartphone app that aims to facilitate self-management of LBP | Smartphone application | selfBACK | Educational content  Physical activity exercises  Behaviour change theory exercises  Artificial intelligent driven plans | 3-months  New plans offered weekly |
| Tonga et al, 2021, Turkey [67] | To support the delivery of hand exercises for patients with rheumatoid arthritis | Smartphone application |  | Hand exercises  Pain tracking and monitoring  Exercise diary  Educational resource  Behaviour change motivational messages | 6-weeks  As often as necessary |
| Tonkin-Crine et al, 2013, United Kingdom [68] | To facilitate CBT self-management of symptoms of IBS | Website | Regul8 | CBT techniques  Symptom tracking  Goal setting | 6-weeks  8 sessions |
| Van Der Meer et al, 2022, Netherlands [69] | To assist with symptom management of TMD complaints | Web-based programme | Physitrack | Exercise programme  Pain monitoring  Chat function | Daily use |
| Whitney et al, 2018, USA [70] | Tracking of chronic pain symptoms over time while also providing graphical summaries. | Smartphone application | Trialist app | Pain tracking  Sleep tracking  Thinking problems  Drowsiness and fatigue tracking  Constipation tracking  Treatment comparisons | 12-months |
| Yeh et al, 2022, USA [71] | A smartphone app as a self-guided tool to help people learn and self-administer APA to manage cLBP | Smartphone application | APA app | Auricular point acupressure Informational content | 1-month  3 times daily for 3 minutes each time |
| Zuidema et al, 2019, Netherlands [72] | To promote self-management techniques and induce behavioural change using intervention mapping framework and the theory of planned behaviour in patients with rheumatoid arthritis | Online programme |  | Educational modules  Pain diary  Activity tracking  Medication tracking  Boundary setting | 12 months |

APA – auricular point acupressure; CBT – cognitive behavioural therapy; cLBP – chronic lower back pain; e-Health – electronic-health; EMA – ecological momentary assessment; HCP – healthcare professional; IBS – irritable bowel syndrome; IPRPs – Interdisciplinary pain rehabilitation programs; OA – osteoarthritis; PCST – pain coping skills training; RA – rheumatoid arthritis; SMS – short messaging service; TMD - temporomandibular disorder; VA – virtual assistant.

**Reference list:**

44. Algeo N, Hunter D, Cahill A, Dickson C, Adams J. Usability of a digital self-management website for people with osteoarthritis: A UK patient and public involvement study. International Journal of Therapy & Rehabilitation. 2017;24(2):78-82. PMID: 121447740. Language: English. Entry Date: 20170228. Revision Date: 20170303. Publication Type: Article. doi: 10.12968/ijtr.2017.24.2.78.

45. Arensman R, Kloek C, Pisters M, Koppenaal T, Ostelo R, Veenhof C. Patient Perspectives on Using a Smartphone App to Support Home-Based Exercise During Physical Therapy Treatment: Qualitative Study. JMIR Human Factors. 2022 2022;9(3). PMID: rayyan-562659184. doi: doi:10.2196/35316.

46. Austin L, Sharp CA, van der Veer SN, Machin M, Humphreys J, Mellor P, et al. Providing 'the bigger picture': Benefits and feasibility of integrating remote monitoring from smartphones into the electronic health record. Rheumatology (United Kingdom). 2020;59(2):367-78. PMID: 632830193.

47. Ball E, Newton S, Rohricht F, Steed L, Birch J, Dodds J, et al. MHealth: providing a mindfulness app for women with chronic pelvic pain in gynaecology outpatient clinics: qualitative data analysis of user experience and lessons learnt. BMJ Open. 2020 2020-3-12;10. PMID: rayyan-562659189.

48. Bostrom K, Varsi C, Eide H, Borosund E, Kristjansdottir OB, Schreurs KMG, et al. Engaging with EPIO, a digital pain self-management program: a qualitative study. BMC health services research. 2022 2022-4-29;22:577. PMID: rayyan-562659211.

49. Cronström A, Dahlberg LE, Nero H, Ericson J, Hammarlund CS. 'I would never have done it if it hadn't been digital': a qualitative study on patients' experiences of a digital management programme for hip and knee osteoarthritis in Sweden. BMJ Open. 2019 May 24;9(5):e028388. PMID: 31129601. doi: 10.1136/bmjopen-2018-028388.

50. Garrett B, Taverner T, McDade P. Virtual Reality as an Adjunct Home Therapy in Chronic Pain Management: An Exploratory Study. JMIR Medical Informatics. 2017;5(2):e11. PMID: 28495661.

51. Godziuk K, Prado CM, Quintanilha M, Forhan M. Acceptability and preliminary effectiveness of a single-arm 12-week digital behavioral health intervention in patients with knee osteoarthritis. BMC Musculoskeletal Disorders. 2023 2023-12;24. PMID: rayyan-562659279.

52. Hogan TP, Etingen B, McMahon N, Bixler FR, Am L, Wacks RE, et al. Understanding Adoption and Preliminary Effectiveness of a Mobile App for Chronic Pain Management Among US Military Veterans: Pre-Post Mixed Methods Evaluation. JMIR Formative Research. 2022 2022;6(1):e33716. PMID: rayyan-540241597.

53. Jeon YH, Flaherty I, Urban H, Wortley S, Dickson C, Salkeld G, et al. Qualitative Evaluation of Evidence-Based Online Decision Aid and Resources for Osteoarthritis Management: Understanding Patient Perspectives. Arthritis Care and Research. 2019 2019-1;71:46-55. PMID: rayyan-562659323.

54. Kawi J, Yeh CH, Lukkahatai N, Hardwicke RL, Murphy T, Christo PJ. Exploring the Feasibility of Virtually Delivered Auricular Point Acupressure in Self-Managing Chronic Pain: Qualitative Study. Evidence-based Complementary and Alternative Medicine. 2022 2022;2022. PMID: rayyan-562659330.

55. Knoerl R, Bockhoff J, Fox E, Giobbie-Hurder A, Berry DL, Berfield J, et al. Cancer Survivors' Perspectives of Virtual Yoga for Chronic Chemotherapy-Induced Peripheral Neuropathy Pain During the COVID-19 Pandemic. CIN: Computers, Informatics, Nursing. 2022;40(9):641-7. PMID: 159061718. Language: English. Entry Date: 20221003. Revision Date: 20230308. Publication Type: Article. doi: 10.1097/CIN.0000000000000937.

56. Lamper C, Huijnen I, de Mooij M, Koke A, Verbunt J, Kroese M. An eCoach-Pain for Patients with Chronic Musculoskeletal Pain in Interdisciplinary Primary Care: A Feasibility Study. International Journal of Environmental Research & Public Health [Electronic Resource]. 2021;18(21):06. PMID: 34770177.

57. Merolli M, Gray K, Martin-Sanchez F. Patient Participation in Chronic Pain Management Through Social Media: A Clinical Study. Studies in health technology and informatics. 2016 2016;225:577-81. PMID: rayyan-562659379.

58. Muehlensiepen F, May S, Hadaschik K, Vuillerme N, Heinze M, Grahammer M, et al. Digitally supported shared decision-making and treat-to-target in rheumatology: a qualitative study embedded in a multicenter randomized controlled trial. Rheumatology International. 2023 2023-4;43:695-703. PMID: rayyan-562659390.

59. Nelligan RK, Hinman RS, Teo PL, Bennell KL. Exploring attitudes and experiences of people with knee osteoarthritis toward a self-directed ehealth intervention to support exercise: Qualitative Study. JMIR Rehabilitation and Assistive Technologies. 2020 2020;7(2). PMID: rayyan-562659401. doi: doi:10.2196/18860.

60. Nordin C, Michaelson P, Eriksson MK, Gard G. It's About Me: Patients' Experiences of Patient Participation in the Web Behavior Change Program for Activity in Combination With Multimodal Pain Rehabilitation. Journal of medical Internet research. 2017 2017-1-18;19:e22. PMID: rayyan-562659407.

61. Rini C, Vu MB, Lerner H, Bloom C, Carda-Auten J, Wood WA, et al. A qualitative study of patient and provider perspectives on using web-based pain coping skills training to treat persistent cancer pain. Palliative & supportive care. 2018 2018-4-1;16:155-69. PMID: rayyan-562659444.

62. Schlett C, Röttele N, van der Keylen P, Schöpf-Lazzarino AC, Klimmek M, Körner M, et al. The Acceptance, Usability, and Utility of a Web Portal for Back Pain as Recommended by Primary Care Physicians: Qualitative Interview Study with Patients. JMIR Formative Research. 2022 2022;6(12). PMID: rayyan-562659458. doi: doi:10.2196/38748.

63. Shewchuk B, Green LA, Barber T, Miller J, Teare S, Campbell-Scherer D, et al. Patients' use of mobile health for self-management of knee osteoarthritis: Results of a 6-week pilot study. JMIR Formative Research. 2021 2021;5(11). PMID: rayyan-562659468. doi: doi:10.2196/30495.

64. Stern BZ, Pila S, Joseph LI, Rothrock NE, Franklin PD. Patients' perspectives on the benefits of feedback on patient-reported outcome measures in a web-based personalized decision report for hip and knee osteoarthritis. BMC Musculoskeletal Disorders. 2022 2022-12;23. PMID: rayyan-562659485.

65. Svanholm F, Turesson C, Löfgren M, Björk M. Acceptability of the eHealth Intervention Sustainable Worker Digital Support for Persons With Chronic Pain and Their Employers (SWEPPE): Questionnaire and Interview Study. JMIR Hum Factors. 2023 Sep 28;10:e46878. PMID: 37768708. doi: 10.2196/46878.

66. Svendsen MJ, Nicholl BI, Mair FS, Wood K, Rasmussen CDN, Stochkendahl MJ. One size does not fit all: Participants' experiences of the selfBACK app to support self-management of low back pain-a qualitative interview study. Chiropractic and Manual Therapies. 2022 2022-12;30. PMID: rayyan-562659488.

67. Tonga E, Williamson E, Srikesavan C, Özen T, Sarıtaş F, Lamb SE. A hand exercise mobile app for people with rheumatoid arthritis in Turkey: design, development and usability study. Rheumatology International. 2021;41(6):1151-60. doi: 10.1007/s00296-021-04860-0.

68. Tonkin-Crine S, Bishop FL, Ellis M, Moss-Morris R, Everitt H. Exploring patients' views of a cognitive behavioral therapy-based website for the self-management of irritable bowel syndrome symptoms. Journal of medical Internet research. 2013 2013;15:e190. PMID: rayyan-562659499. doi: doi:.

69. van der Meer HA, de Pijper L, van Bruxvoort T, Visscher CM, Nijhuis-van der Sanden MWG, Engelbert RHH, et al. Using e-Health in the physical therapeutic care process for patients with temporomandibular disorders: a qualitative study on the perspective of physical therapists and patients. Disability and rehabilitation. 2022 01 Feb;44(4):617-24. PMID: 632104005.

70. Whitney RL, Ward DH, Marois MT, Schmid CH, Sim I, Kravitz RL. Patient perceptions of their own data in mhealth technology–enabled N-of-1 trials for chronic pain: Qualitative study. JMIR mHealth and uHealth. 2018;6(10). doi: 10.2196/10291.

71. Yeh CH, Kawi J, Ni A, Christo P. Evaluating Auricular Point Acupressure for Chronic Low Back Pain Self-Management Using Technology: A Feasibility Study. Pain Management Nursing. 2022;23(3):301-10. PMID: 157501161. Language: English. Entry Date: 20220701. Revision Date: 20220701. Publication Type: Article. doi: 10.1016/j.pmn.2021.11.007.

72. Zuidema RM, Van Dulmen S, Nijhuis-van der Sanden MWG, Fransen J, Van Gaal BGI. qLessons learned from patients with access to an online self-management enhancing program for RA patients: Qualitative analysis of interviews alongside a randomized clinical trial. Patient Educ Couns. 2019 Jun;102(6):1170-7. PMID: 30638903. doi: 10.1016/j.pec.2019.01.005.

73. Östlind E, Ekvall Hansson E, Eek F, Stigmar K. Experiences of activity monitoring and perceptions of digital support among working individuals with hip and knee osteoarthritis – a focus group study. BMC Public Health. 2022;22(1). doi: 10.1186/s12889-022-14065-0.

74. Overton M, Swain N, Falling C, Gwynne-Jones D, Fillingim R, Mani R. Experiences and Perceptions of Using Smartphone Ecological Momentary Assessment for Reporting Knee Osteoarthritis Pain and Symptoms. Clinical Journal of Pain. 2023 19 Sep;39(9):442-51. PMID: 2026479267.

75. De Groef A, Evenepoel M, Van Dijck S, Dams L, Haenen V, Wiles L, et al. Feasibility and pilot testing of a personalized eHealth intervention for pain science education and self-management for breast cancer survivors with persistent pain: a mixed-method study. Supportive Care in Cancer. 2023 2023-2;31. PMID: rayyan-562659237. doi: doi:.

76. Seppen BF, Wiegel J, Nurmohamed MT, Bos WH, ter Wee MM. Facilitators and barriers to adhere to monitoring disease activity with ePROs: a focus group study in patients with inflammatory arthritis. Rheumatology International. 2023 2023-4;43:677-85. PMID: rayyan-562659462. doi: doi:.

77. Garrett BM, Tao G, Taverner T, Cordingley E, Sun C. Patients perceptions of virtual reality therapy in the management of chronic cancer pain. Heliyon. 2020 2020;6(5). PMID: rayyan-562659274. doi: doi:10.1016/j.heliyon.2020.e03916.

78. Grolier M, Arefyev A, Pereira B, Tavares Figueiredo I, Gerbaud L, Coudeyre E. Refining the design of a smartphone application for people with chronic low back pain using mixed quantitative and qualitative approaches. Disability and rehabilitation. 2023 2023-2-1:145-50. PMID: rayyan-562659290.

79. Hoving JL, Zoer I, Van Der Meer M, Van Der Straaten Y, Logtenberg-Rutten C, Kraak-Put S, et al. E-health to improve work functioning in employees with rheumatoid arthritis in rheumatology practice: A feasibility study. Scandinavian Journal of Rheumatology. 2014 01 Nov;43(6):481-7. PMID: 600693450.

80. Seppen BF, Wiegel J, L'Ami M J, Duarte Dos Santos Rico S, Catarinella FS, Turkstra F, et al. Feasibility of Self-Monitoring Rheumatoid Arthritis With a Smartphone App: Results of Two Mixed-Methods Pilot Studies. JMIR Formative Research. 2020;4(9):e20165. PMID: 32955447.
