# Supplementary material for: Patients’ Experiences of Digital Health Interventions for the Self-Management of Chronic Pain: Systematic Review and Thematic Synthesis
Source: J Med Internet Res. 2025 Mar 18;27:e69100. doi: 10.2196/69100 (PMC11962327; doi:10.2196/69100)
Supplement: Multimedia Appendix 7 [file jmir_v27i1e69100_app7.doc]

Multimedia Appendix 7 –Quotes from the included studies illustrating each theme and subtheme identified within the data.

| **Themes** | **Subthemes** | **Participant quotations** |
| --- | --- | --- |
| **Personal growth** | Gaining new insights | *“You may think “Oh my God, this is something wrong with me seriously” and then make the symptoms worse. Um, so I think having that actually on the Internet site it made me stop and reflect, and sometimes I think stop me from running ahead of myself”* (p.9) [58].  “*It has given me exercises to help reduce stress a bit. To let things go, that it’s not so important, perhaps, and to breathe with your stomach*” (p.7) [38].  “*I had read about CBT, but I had never thought of it as a help for my condition*” (p.5) [50].  *“It was really for me was just putting it in black and white, what I needed to do, which in some ways, it made you think a bit more, maybe took yourself out of the situation and made you think about things… more of an insight into what was going on”* (p.8) [58].  “*I have learned various breathing exercises and relaxation methods that I was not familiar with before. And I have learned that you have to take the time, in a stressful everyday life. Even if you are at home, sick, and in pain, you have to prioritize yourself*” (p.9) [38].  “*I found that that made my pain much worse, and I think part way through I actually stopped doing it and went to a different one to do my 30 min a day, because the pain was just so aggravated by concentrating on it”* (p.6) [67]. |
|  | Renewed mindset | “*I could see the change that was happening, I was able to speak up for myself…I can’t explain it, even now I am getting emotional…it’s just a lack of focus, I just needed directio*n” [37].  “*I think it was probably reassuring in a way…that obviously the symptoms I’d had were very common and were similar to other people...‘cause I hadn’t really talked to anyone else about it*” (p.8) [58].  “*The ABATON RA app is such a hold for me, it makes me feel calmer. Because I see that [the disease] is slowing down, it’s working, [the medication] is kicking in and it's great and everything's in the green”* (p.699) [48].  “*At home one can see exactly how to do the exercises, uh, because it is explained, but yeah when you are at home you think how should I position my head […] so yes it is convenient to have the app at hand and it is clear in that video how the exercises should be performed exactly […], I think that’s nice, because one forgets it*” (p.7) [59].  “*Yes, because I mean I’m a lot more, happier, and confident. And like I said, I used to walk backwards and forwards [to work] and when I couldn’t, it was a real pain, because you need exercise. And I’m doing that*” (p.52) [43].  *“[My family] saw me helping myself rather than them trying to ﬁgure out how to help me ... When I told them I was going through this program, they said, ‘that’s amazing because we see a change*” (p.162) [51].  “*What is useful with EPIO is to become more ... to achieve a degree of inner peace, in relation to my pain… I will never get rid of my pain … rather than arguing with it all the time, standing on separate sides of a courtyard, fighting, to be a little more in tune. So, becoming better friends with the pain sort of, and work with the pain, not just against it*” (p.9) [38]. |
| **Active involvement** | Motivation | “*You get disquiet if you do not reach 7,000 steps… I think it happened to me one day and that was very tough…”* (p.5) [63].  “*It will be easy to push or trigger yourself to go those steps extra… it is easy to motivate and take another walk to reach the goal*” (p.4) [63].  “*The body size and age of the person demonstrating exercises was relatable, making patients more comfortable engaging with the exercises*” (p.8) [41]. |
|  | Improved access | “*It’s just very easy. You carry your phone with you every day anyway, so when you forget something, you can just open the app and find it; very easy*” (p.5) [35].  “*I think that the program could be very good for a lot of people, and I think online as a whole – because there’s lots of people who can’t get out to a gym or can’t afford a gym – it’s a good idea*” (p.8) [41]. |
|  | Healthcare decision making | *“...my own thinking about my situation was confirmed by the content in the web-program (Web-BCPA)...this made me feel safe to share those thoughts (with the health care professionals) to acquire new knowledge that I can use in meetings with people that are involved in my rehabilitation...I was equipped with putting words on my thinking*” (p.5) [50]. |
| **Connectedness and support** |  | “A*t the end of the primary treatments, contact with them is reduced and becomes very limited. At that moment, you ask yourself the question what would give me more energy and what are the limits of my body. If I had received this program at that moment, I would have worried less about my pain sensations”* (p.14) [65].  *“In the beginning, I found the writing style of the program encouraging to keep going”* (p.9) [65].  “*You feel really connected to other people and supported to do something to help the situation*” (p.579) [47]. |
